# Integrative genomic analysis of early neurogenesis reveals a temporal genetic program for differentiation and specification of preplate and Cajal-Retzius neurons

**DOI:** 10.1371/journal.pgen.1009355

**Published:** 2021-03-24

**Authors:** Jia Li, Lei Sun, Xue-Liang Peng, Xiao-Ming Yu, Shao-Jun Qi, Zhi John Lu, Jing-Dong J. Han, Qin Shen

**Affiliations:** 1 Key Laboratory of Spine and Spinal Cord Injury Repair and Regeneration of Ministry of Education, Orthopaedic Department of Tongji Hospital, School of Life Sciences and Technology, Tongji University, Shanghai, China; 2 PTN graduate program, School of Life Sciences, Peking University, Beijing, China; 3 School of Medicine, Tsinghua University, Beijing, China; 4 PTN graduate program, School of Life Sciences, Tsinghua University, Beijing, China; 5 MOE Key Laboratory of Bioinformatics, Center for Synthetic and Systems Biology, School of Life Sciences, Tsinghua University, Beijing, China; 6 Key Laboratory of Computational Biology, CAS Center for Excellence in Molecular Cell Science, Collaborative Innovation Center for Genetics and Developmental Biology, Chinese Academy of Sciences-Max Planck Partner Institute for Computational Biology, Shanghai Institutes for Biological Sciences, Chinese Academy of Sciences, Shanghai, China; 7 Frontier Science Center for Stem Cell Research, Ministry of Education, School of Life Sciences and Technology, Tongji University, Shanghai, China; 8 Brain and Spinal Cord Clinical Research Center, Tongji University, Shanghai, China; Duke-NUS Medical School, SINGAPORE

## Abstract

Neurogenesis in the developing neocortex begins with the generation of the preplate, which consists of early-born neurons including Cajal-Retzius (CR) cells and subplate neurons. Here, utilizing the Ebf2-EGFP transgenic mouse in which EGFP initially labels the preplate neurons then persists in CR cells, we reveal the dynamic transcriptome profiles of early neurogenesis and CR cell differentiation. Genome-wide RNA-seq and ChIP-seq analyses at multiple early neurogenic stages have revealed the temporal gene expression dynamics of early neurogenesis and distinct histone modification patterns in early differentiating neurons. We have identified a new set of coding genes and lncRNAs involved in early neuronal differentiation and validated with functional assays *in vitro* and *in vivo*. In addition, at E15.5 when Ebf2-EGFP+ cells are mostly CR neurons, single-cell sequencing analysis of purified Ebf2-EGFP+ cells uncovers molecular heterogeneities in CR neurons, but without apparent clustering of cells with distinct regional origins. Along a pseudotemporal trajectory these cells are classified into three different developing states, revealing genetic cascades from early generic neuronal differentiation to late fate specification during the establishment of CR neuron identity and function. Our findings shed light on the molecular mechanisms governing the early differentiation steps during cortical development, especially CR neuron differentiation.

## Author summary

Neural stem cells and progenitor cells in the embryonic brain give rise to neurons following a precise temporal order after initial expansion. Early-born neurons including Cajal-Retzius (CR) cells and subplate neurons form the preplate in the developing cerebral cortex, then CR neurons occupy the layer 1, playing an important role in cortical histogenesis. The molecular mechanisms governing the early neuronal differentiation processes remain to be explored. Here, by genome-wide approaches including bulk RNA-seq, single-cell RNA-seq and ChIP-seq, we comprehensively characterized the temporal dynamic gene expression profile and epigenetic status at different stages during early cortical development and uncovered molecularly heterogeneous subpopulations within the CR cells. We revealed CR neuron signatures and cell type-specific histone modification patterns along early neuron specification. Using *in vitro* and *in vivo* assays, we identified novel lncRNAs as potential functional regulators in preplate differentiation and CR neuron identity establishment. Our study provides a comprehensive analysis of the genetic and epigenetic programs during neuronal differentiation and would help bring new insights into the early cortical neurogenesis process, particularly the differentiation of CR neurons.

## Introduction

The mammalian cortical neurogenesis occurs on a precise time schedule during development. Neural stem cells (NSCs), the neuroepithelial cells and radial glial cells (RGCs) in the ventricular zone (VZ), first undergo symmetric cell divisions to expand, then begin to produce neurons [1,2]. The first-born neurons are generated as early as E10 in mouse and form the preplate or the primordial plexiform layer, which situates above the VZ and beneath the pial surface [1,3,4]. The preplate then split into the marginal zone (MZ, layer 1) and the subplate by immigrating cortical plate neurons around E13.5 in mouse and the seventh to eighth week of gestation in human [5–7], setting up of the upper and lower borders of the cortical plate. The formation and splitting of preplate are the first steps in the establishment of the highly organized cortical layers.

The preplate and its derivatives play a critical role in regulating subsequent inside-out neuronal migration, cortical lamination and the establishment of topographic connections [8]. Subplate neurons contribute to the guidance of corticofugal and thalamocortical axons during early development and are important for the functional maturation and plasticity of the cortical circuitry [9,10]. Cajal-Retzius (CR) neurons are the major cell type in layer 1, the most superficial cortical layer. Discovered more than a century ago by Retzius and Ramon y Cajal, CR neurons have been studied extensively [4,11]. Best known for their expression of *Reln*, CR neurons regulate the inside-out migration of cortical neurons, correct layer formation, maintenance of RGC and cortical patterning [12]. Loss of *Reln* or mislocalization of CR cells contributes to defective migration and disordered cortical layer formation that are associated with human brain malformation such as lissencephaly [13] and dystroglycanopathy [14]. Although the functional importance of these early differentiating neurons is well recognized, characterization of cell-intrinsic, dynamic gene expression patterns in these cells remain incomplete. Recent studies have identified the genes enriched in subplate neurons by microarray and RNA-seq analysis [15–17], providing a comprehensive gene expression profile of the subplate at different developmental stages, which offers valuable insights on molecular mechanisms underlying neurodevelopmental disorders [18]. However, how gene expression is dynamically regulated during the transition from the preplate to CR neurons remains unclear.

We previously have shown that in the Ebf2-EGFP transgenic mice (GENSAT project), the GFP signal is expressed specifically in early differentiating preplate neurons and persistent in CR cells [19–21]. After preplate splitting, virtually all of the EGFP-positive cells exhibit morphological and molecular features of CR neurons in the embryonic and postnatal brain, consistent with the pattern of the *Ebf2*^*GFPiCre*^ [19,22], making this line suitable for tracing the development and profiling genetic and epigenetic pattern of early-born neurons including CR neurons. In addition, ablation and fate mapping studies have revealed diverse origins other than cortical neuroepithelium for CR neurons, such as cortical hem, septum, ventral pallium, which could be recognized by specific molecular markers [23,24]. However, whether CR cells from different origins maintain their distinct regional identities after they reach the layer 1 in the neocortex remains to be addressed.

Single-cell gene expression profiling allows further dissection of the heterogeneity and developmental trajectory within a cell population [25,26]. While multiple types of neurons in adult cortex and hippocampus have been identified by single-cell RNA-sequencing [26,27], a full characterization of early differentiating neurons at single-cell level has not been done before. In this study, we used FACS-purified Ebf2-EGFP-expressing cells to probe the dynamic molecular mechanisms of preplate and CR neurons during the early stages of forebrain development. Whole genome transcriptome analysis identified the initial differences between the preplate and progenitor cells at E11.5, reflecting the genetic choice during the first cell fate decision in neural differentiation. We also recognized clusters of genes that displayed temporal changes corresponding to different developmental trajectories. Genome-wide active (H3K27ac) and repressive (H3K27me3) histone modifications in preplate neurons, CR neurons and NPCs revealed that occupancies of histone marks around TSS and distal regions were distinct between different cell types, and unique histone modification pattern emphasized on promoter regions to reinforce CR neuron specification, suggesting an epigenetic role in acquisition and maintenance of cell type-specific identities. By bioinformatic functional assessments, we also identified and validated the functions of lncRNAs that were enriched in CR neurons during neuronal differentiation. Furthermore, we identified novel subpopulations of CR neurons from the E15.5 neocortex using single-cell sequencing and uncovered distinct developmental states within the pure population. Analysis of CR neuron lineage revealed molecular cascades along generic neuronal differentiation to CR cell-specific fate determination.

## Results

### Characterization of Ebf2-EGFP-expressing cells in the early cerebral cortex

**Fig 1:**
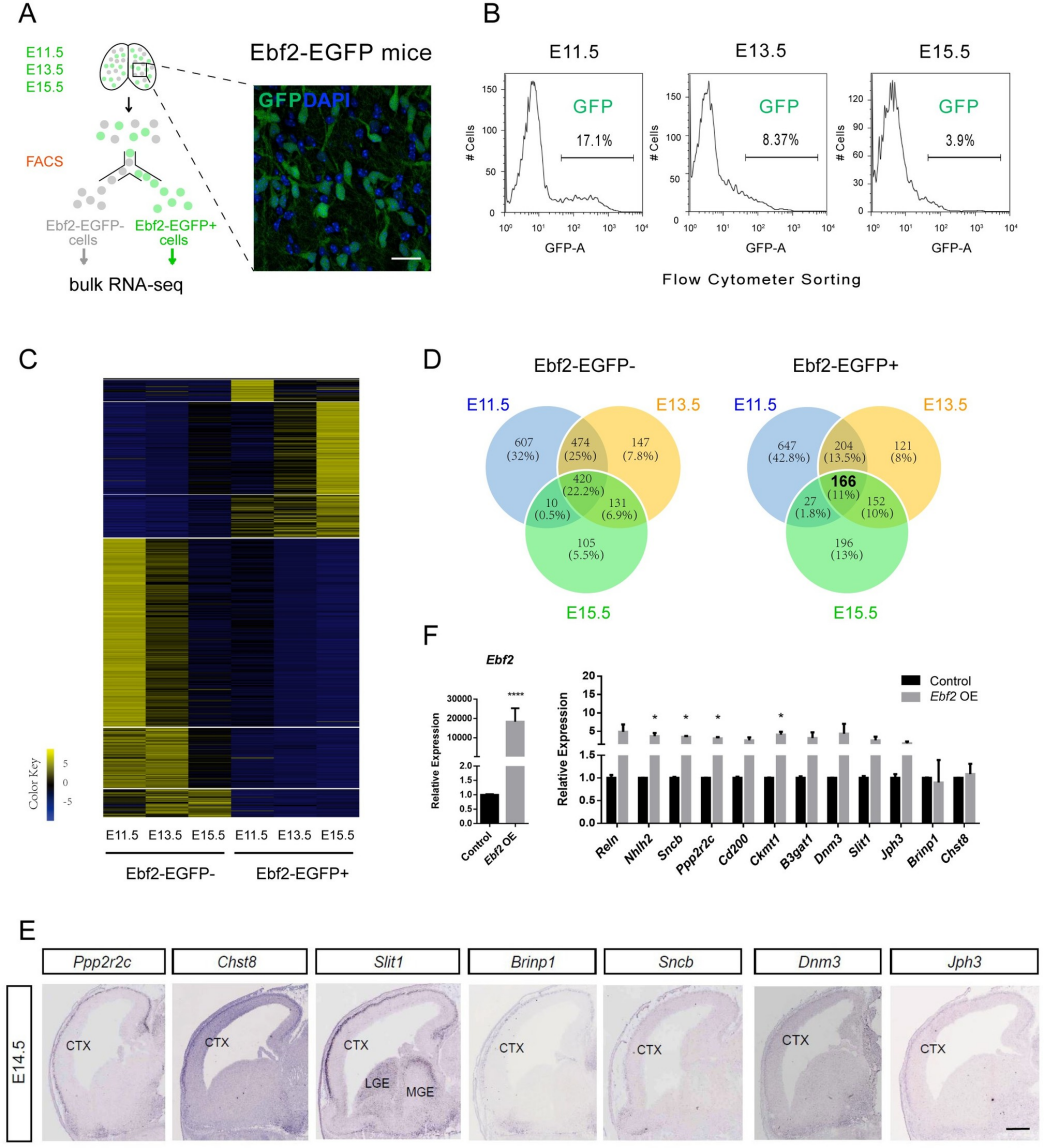
Transcriptome sequencing of Ebf2-EGFP+ and Ebf2-EGFP- cells in the early embryonic stage. (A) Schematic illustration of FACS purification of Ebf2-EGFP+ and Ebf2-EGFP- cells from early embryonic developmental stages E11.5, E13.5 and E15.5, respectively. (B) The percentages of Ebf2-EGFP+ cells collected from FACS at each stage are calculated and shown in the graph. (C) Heatmap of all differential expressed genes (FDR<0.05) in Ebf2-EGFP+ & Ebf2-EGFP- samples at the three early embryonic stages are shown. (D) Venn diagrams displaying the extent of overlapping between DEGs at the three embryonic stages. (E) Expression of 7 CR-specific coding genes are found to be selectively expressed in cortical layer 1 of E14.5 mouse embryos (*In situ* hybridization images from GenePaint.org). Scale bar, 650 μm. Another CR-specific coding gene *Nhlh2* is shown in the S1D Fig. (F) qRT-PCR analysis comparing the expression levels of CR-specific genes in primary neural cell culture assay after virus infection with control (H1) or *Ebf2*-overexpression. Data represent mean ± SEM (n = 3 independent experiments, *P<0.05, Student’s T test).

To investigate the molecular mechanisms and temporal dynamic gene network during the early stages of neural differentiation, we performed bulk RNA-seq of Ebf2-EGFP+ and Ebf2-EGFP- cells at multiple early neurogenic time points as E11.5, E13.5 and E15.5. Previous studies have provided comprehensive gene profiles and analysis of temporal transcriptomic changes of subplate neurons [15,17,18], hence here we focused on the CR neurons. In addition, we previously have shown that at E11.5, Ebf2-EGFP+ cells are mostly preplate neurons including both subplate neurons and CR neurons. At E13.5, Ebf2-EGFP expression is diminishing in subplate neurons but maintains in CR neurons. By E15.5, Ebf2-EGFP+ cells are mostly CR neurons [19]. Therefore, in this study we purified Ebf2-EGFP-expressing cells by FACS from the embryonic mouse forebrain at E11.5, E13.5 and E15.5, to compare the developmental gene expression dynamics during preplate formation and CR neurons differentiation (Fig 1A). At E11.5, Ebf2-expressing cells consisted of 17.10% ± 1.07% of the total cells. At E13.5 and E15.5, the percentage of Ebf2-expressing cells gradually decreased to 8.37 ± 2.29% and 3.90 ± 1.59%, respectively (Fig 1B). 100% of FACS-sorted Ebf2-EGFP+ cells were β-III tubulin+ at three stages, indicating these cells have committed neuronal fate decision, consistent with our previous *in vivo* analysis [19]. Accordingly, after culturing sorted and unsorted cells for 4 days, almost all of the Ebf2-EGFP+ cells maintained single and extended processes, while the Ebf2-EGFP- cells proliferated and generated large clones similar to the unsorted cell group, indicating a good separation of differentiating neurons from progenitor cells (S1A Fig).

### Identification of Ebf2-EGFP+ cell-enriched genes in the developing mouse forebrain

Next, we collected FACS-selected Ebf2-EGFP+ cells and Ebf2-EGFP- cells from the three neurogenic stages and isolated total RNA for whole-genome transcriptome sequencing. Two biological replicates for each cell type (Ebf2-EGFP+ and Ebf2-EGFP- cells) were sequenced and their expressed transcripts were identified as described in Methods. To verify the reproducibility of our samples, we analyzed the Pearson’s correlation coefficient (Pearson’s r) for the replicates and there was a strong association between different preparations/batches of FACS-sorted cells from the same stage (S1B Fig). To validate the RNA-seq results, we selected several coding genes, including CR neuron markers (*Ebf2* and *Calb2*) and genes associated with CR neurons fate regulation (*Foxg1*, *Tbr1*, *Ebf1*, and *Ebf3*) [11,19,23,28–30], and verified by qPCR. The results showing the temporal dynamic expression levels of these genes were in accordance with that detected by RNA-seq (S1C Fig). Furthermore, the temporal expression trends were consistent with the mRNA expression pattern by *in situ* hybridization (ISH) from the data available in Allen Brain Atlas (S1C Fig) [31].

We used DE-Seq to analyze the dynamic expression profiles during the early cortical differentiation period. 4859 genes exhibited significant differences in expression level between the two cell populations during the time courses and were selected as DEGs for further analysis (Fig 1C and S1 Table). We analyzed DEGs expressed in any of the developmental stages sequenced (E11.5, E13.5 and E15.5). Genes with expression level (FPKM) more than 1, and 2-fold higher in Ebf2-EGFP+ population than that in Ebf2-EGFP- population were defined as Ebf2-EGFP+ cell-enriched genes, CR-enriched genes in short. We found that there were 1044 genes at E11.5, 643 genes at E13.5, and 541 at E15.5 enriched in Ebf2-EGFP+ cells, respectively. Considerable overlaps were found between the different stages (Fig 1D). In total, 1513 genes displayed enriched expression in Ebf2-EGFP+ population at least at one stage, and 1894 such genes in Ebf2-EGFP- population. Of these, 166 genes were consistently enriched in Ebf2-EGFP+ population throughout all three embryonic stages, and 420 genes were consistently enriched in Ebf2-EGFP- population (Fig 1D). The 166 DEGs included known CR neuron markers such as *Reln* and *Trp73*, and genes known to be expressed in CR neurons such as *Calb2*, *Tbr1*, *Ebf3*, *Cdkn1a*. We further defined the DEGs with FPKM ≥ 5 in Ebf2-EGFP+ cells, and fold change ≥ 2 at all three stages as CR-specific genes. After excluding 10 subplate genes [17,32] and 14 CR genes reported in previous studies [23], 77 genes were considered novel candidates associated with CR neurons as we have not found reported association of these genes with CR neurons in the literature. With references to the public ISH imaging database (Allen Brain Atlas and Eurexpress)[31], we were able to confirm the mRNA expression pattern of 56 genes among the 77 CR-specific genes in the layer 1 of the developing cerebral cortex, where cortical CR neurons resided. We also noticed that there were genes showing high expression levels in both Ebf2-EGFP+ cells and Ebf2-EGFP- cells although their expression levels met the fold change criteria. To obtain the genes specifically expressed in Ebf2-EGFP+ cells but not in Ebf2-EGFP- cells, we added a cutoff of FPKM ≤ 15 in Ebf2-EGFP- cells considering that CR-specific genes should be expressed at low level in Ebf2-EGFP- cells at these stages. 33 genes remained after the filtering and we confirmed that 8 with specific expression in layer 1 cells: the 8 genes are *Nhlh2*, *Ppp2r2c*, *Chst8*, *Slit1*, *Brinp1*, *Sncb*, *Dnm3*, *Jph3*, belonging to transcription factors, synapse molecules, and axon guidance molecules (Figs 1E and S1D and S1E and S1 Table). Hence, these genes could serve as CR-specific markers for future studies.

Previous studies have shown that *Ebf2* marked CR neurons and regulated the generation of this cell population in the cerebral cortex layer 1 [19,21–23], so we hypothesized that genes responding to *Ebf2* overexpression or knockdown were likely to be correlated with CR neuron regulation. Remarkably, the expressions of these 8 genes were affected by overexpression or knockdown of *Ebf2* (Figs 1F and S1E). For example, *Sncb*, *Ckmt1* were significantly upregulated or inhibited by overexpression and knockdown of *Ebf2*, respectively. *Nhlh2*, *Ppp2r2c* responded to *Ebf2* overexpression but not knockdown, the expression of *Slit1* and *B3gat1* were decreased by *Ebf2* knockdown (Figs 1F and S1E). These genes were all involved in cell proliferation, differentiation and migration processes [33–38], so they might also participate in CR neuron generation and differentiation during early neurogenic stage. Specifically, similar to *Ebf2*, *Nhlh2* also played a role in neuroendocrine and controled GnRH neuron migration [35], so it could be possible that *Nhlh2* might be involved in *Ebf2* signaling pathway in regulating CR neurons.

Cortical CR neurons are considered to have multiple extra-cortex origins [39]. We and others have shown previously that *Foxg1* suppressed cortical CR neuron fate [28,29], therefore we hypothesized that CR-specific genes identified by our RNA-seq analysis could be regulated by *Foxg1* expression. Indeed, silencing *Foxg1* using shRNA up-regulated the mRNA levels of *Ebf2*, *Reln*, and CR-specific genes (*Nhlh2*, *Ppp2r2c* and *Cd200*) (S1F Fig).

**Fig 2:**
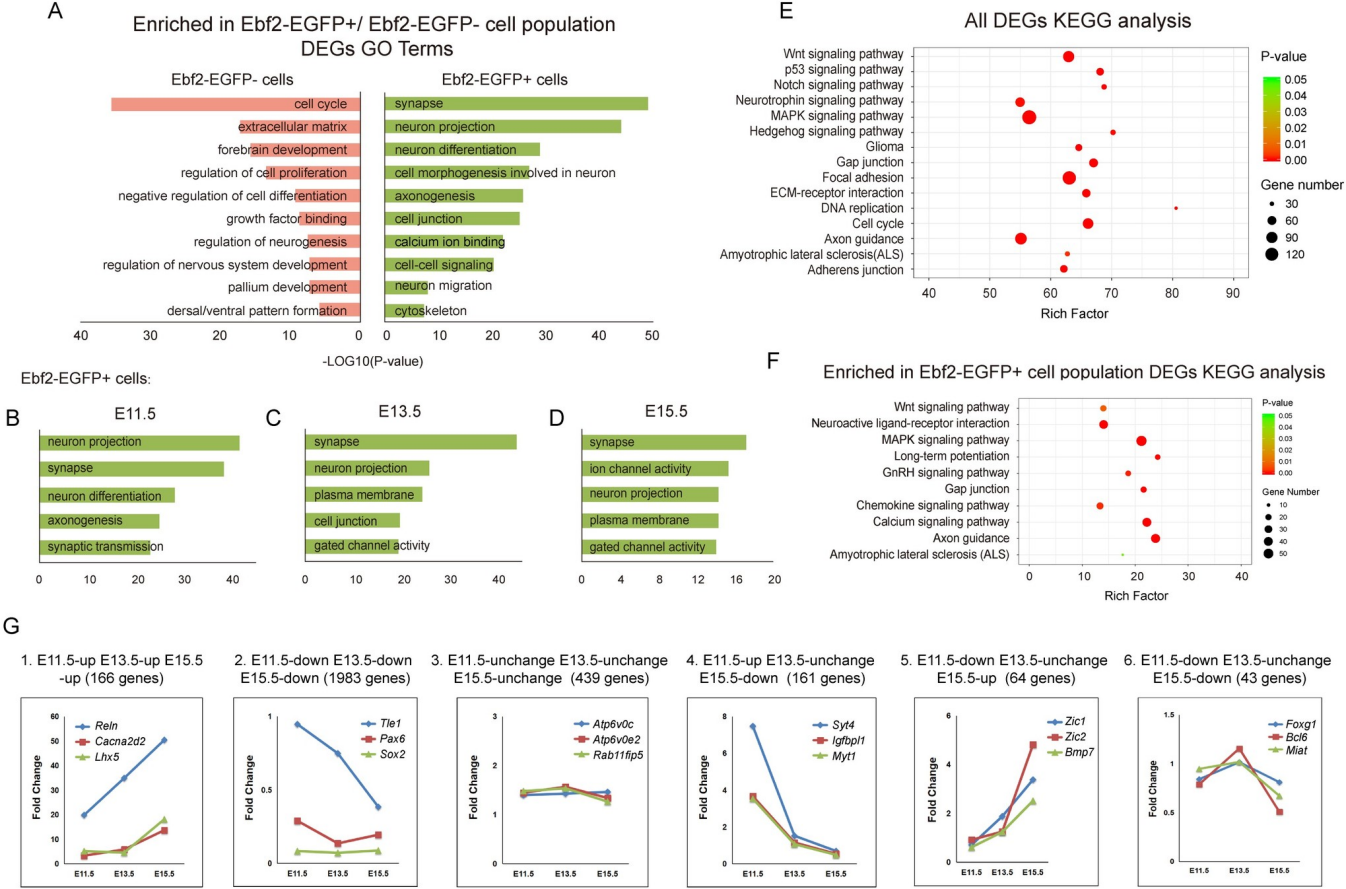
Temporal dynamic DEGs in Ebf2-EGFP+ cells and Ebf2-EGFP- cells show early neuron differentiation progression. (A) Enriched functional assessments (including biological processes, cellular components and molecular function) of highly expressed DEGs (fold change ≥ 2) in Ebf2-EGFP+ or Ebf2-EGFP- cell population. (B-D) Enriched functional assessments of highly expressed DEGs in Ebf2-EGFP+ cell population at E11.5, E13.5 or E15.5, respectively. (E-F) Significantly (p<0.001, FDR<0.001) enriched KEGG pathways within DEGs from all RNA-seq samples and DEGs enriched in Ebf2-EGFP+ samples, respectively. (G) Temporal dynamic expression patterns of DEGs across the three different early neurogenic stages (y axis = Fold Change between Ebf2-EGFP+ cells and Ebf2-EGFP- cells). Noted the dynamic pattern is related to functions of associated genes.

We performed Gene Ontology analysis using the DAVID program to predict the potential functions of DEGs that were enriched in the Ebf2-EGFP+ and the Ebf2-EGFP- cell population, respectively (Fig 2A–2D and S2 Table). The GO terms associated with neuronal differentiation and functions were highly enriched in the DEGs expressed in the Ebf2-EGFP+ cell population, including “synapse”, “neuron projection”, “neuron differentiation”, “cell morphogenesis involved in neuron”, “axonogenesis” and “neuron migration” (Fig 2A). In contrast, the GO terms enriched in the Ebf2-EGFP- cell population were mostly correlated with progenitor cell properties, such as “forebrain development”, “regulation of cell proliferation”, “growth factor binding”, “regulation of neurogenesis” and “dorsal/ventral pattern formation” (Fig 2A).

In addition, we utilized the KEGG pathway repository to help determine the relative representation of specific pathways in our global RNA-seq expression data. The results showed that significantly enriched KEGG pathways of the global DEGs pointed out many signaling pathways implicated in normal neural development (Fig 2E and S2 Table). For example, genes falling under “MAPK Signaling Pathway”, “Wnt Signaling Pathway” and “DNA replication” were enriched. The “Focal Adhesion” and “Axon Guidance” pathways were also enriched, indicating that regulation of cell morphology and neuronal differentiation were involved. Moreover, we performed the KEGG pathway analysis focusing on the DEGs enriched in Ebf2-EGFP+ cell population (Fig 2F and S2 Table). As expected, the major groups of KEGG categories enriched in this data set were “Calcium Signaling Pathway”, “Axon Guidance”, “Neuroactive Ligand-receptor Interaction”, “Gap Junction”, “GnRH Signaling Pathway”, signifying neuronal differentiation properties. Interestingly, as *Ebf2* is an important factor in GnRH neuron differentiation [40], the enrichment of GnRH Signaling pathway suggests that it would regulate similar pathway genes in cortical neurons.

### Temporal transcriptome analysis of Ebf2-EGFP+ cells reveals early neuronal specification and CR neuron differentiation dynamics

The high enrichment of these GO analysis terms and KEGG pathways imply the transcriptional differences between the two cell populations are consistent with their different cell type identities. The components of Ebf2-EGFP+ and Ebf2-EGFP- cell population change as development proceeds. Ebf2-EGFP+ cells are mainly preplate neurons including both subplate and CR neurons at E11.5, while Ebf2-EGFP+ cells are purely CR neurons by E15.5 [19]. In contrast, at E11.5 most if not all of the Ebf2-EGFP- cells are progenitor cells while at E13.5 and E15.5 the Ebf2-EGFP- population also include cortical plate neurons. Therefore, the genes that are highly enriched in Ebf2-EGFP+ cells at all three stages, particularly those with increased enrichments, could be specifically involved in CR neuron differentiation, while the genes that are highly enriched in Ebf2-EGFP+ cells at E11.5 but not at E13.5 or E15.5 could be involved in regulating general neuronal differentiation processes. Hence, we further compared temporal changes in global DEGs at the three different stages. Genes with expression level (FPKM) in Ebf2-EGFP+ population 2 folds higher than that in Ebf2-EGFP- population were defined as “up-regulated”. If the ratio of FPKM (Ebf2-EGFP+/ Ebf2-EGFP-) was between 2 and 1, these genes were defined as “unchanged”. If the ratio was between 1 and 0, these genes were defined as “down-regulated”. Theoretically, we expected 27 possible combinations of expression change patterns over the three time points, while our analysis showed 26 classes, missing the pattern of “E11up—E13down—E15up”. These 26 classes could be classified into 6 groups representing various aspects of early neurogenesis, and 6 typical classes were shown in Figs 2G and S2 and S3 Table. We hypothesized that CR-specific genes were consistently higher at the three stages, while the difference in genes involved in global neuronal differentiation would no longer be evident after E13.5 as the Ebf2-EGFP- population at this stage also included newly generated cortical plate neurons. Indeed, as expected, the genes in the group “E11up—E13up—E15up” included the well-known CR marker genes, such as *Reln*, *Ebf2*, *Trp73* and *Lhx5* [19,22,41], and mature neuronal genes related to axon regeneration such as *Cacna2d2* [42]. In contrast, genes in the “E11down—E13down—E15down” maintained higher expression in Ebf2-EGFP- cells, showing that they might function in regulating NSC proliferation, such as *Sox2*, *Pax6* and *Hes5* [43,44]. Genes that are first expressed in the progenitor cells then in cortical neurons other than preplate and CR neurons may also be included in this group, such as *Tle1* [45]. The “E11up—E13unchange—E15down” group included the subplate genes identified previously, such as *Syt4*, *Igfbpl1*, *Myt1* [17,32], reflecting that subplate genes were highly expressed in the preplate on E11.5, and with decreased expression in layer1 when the preplate splitted later on E13.5 and E15.5. In addition, it also contained genes responsible for radial migration of neurons including *Rnd2* and *Plxna4* [46,47]. Interestingly, looking up in the public *In Situ* Hybridization image resources such as Allen Brain Atlas, we found particular expression patterns of the 22 genes in the “E11down—E13unchange—E15up” group. 77.3% (17/22) of these genes initially had higher expression in the cortical hem or the medial ganglionic eminence (MGE) at E11.5, then appeared in layer 1 at E13.5 and E15.5, implying that these genes might be involved in CR neurons migration from various origins. For example, *Zic1* and *Zic2* were expressed in the progenitor cells in the septum and cortical hem, and *Bmp7* was expressed in the cortical hem, meninges, and choroid plexus. These were all typical CR neuron origins [48,49]. The genes in the “E11down—E13unchange—E15down” group included *Foxg1*, *Bcl6* and *Miat*, which were expressed along the lineage of neural progenitor cells [50,51]. The “E11unchange—E13unchange—E15unchange” group was enriched with genes associated with basic cellular organelles or activities that required for various eukaryotic cells. For example, *Atp6v0c* and *Atp6v0e2* in this group were vacuolar ATPase and played essential roles in maintaining the PH value in the cellular compartments, and during protein degradation [52,53]. *Rab11fip5* is reported to participate in protein trafficking between different organelle membranes [54]. We also performed qPCR tests to validate the temporal dynamics of these gene expressions and obtained generally consistent results (S1C and S2B Figs). Taken together, our analysis reveals that temporal gene expression dynamics reflects distinct developmental processes, allowing us to identify novel factors that play specific roles in early cortical neurogenesis and CR development.

### Identification of potential functional lncRNAs enriched in Ebf2-EGFP+ cells

**Fig 3:**
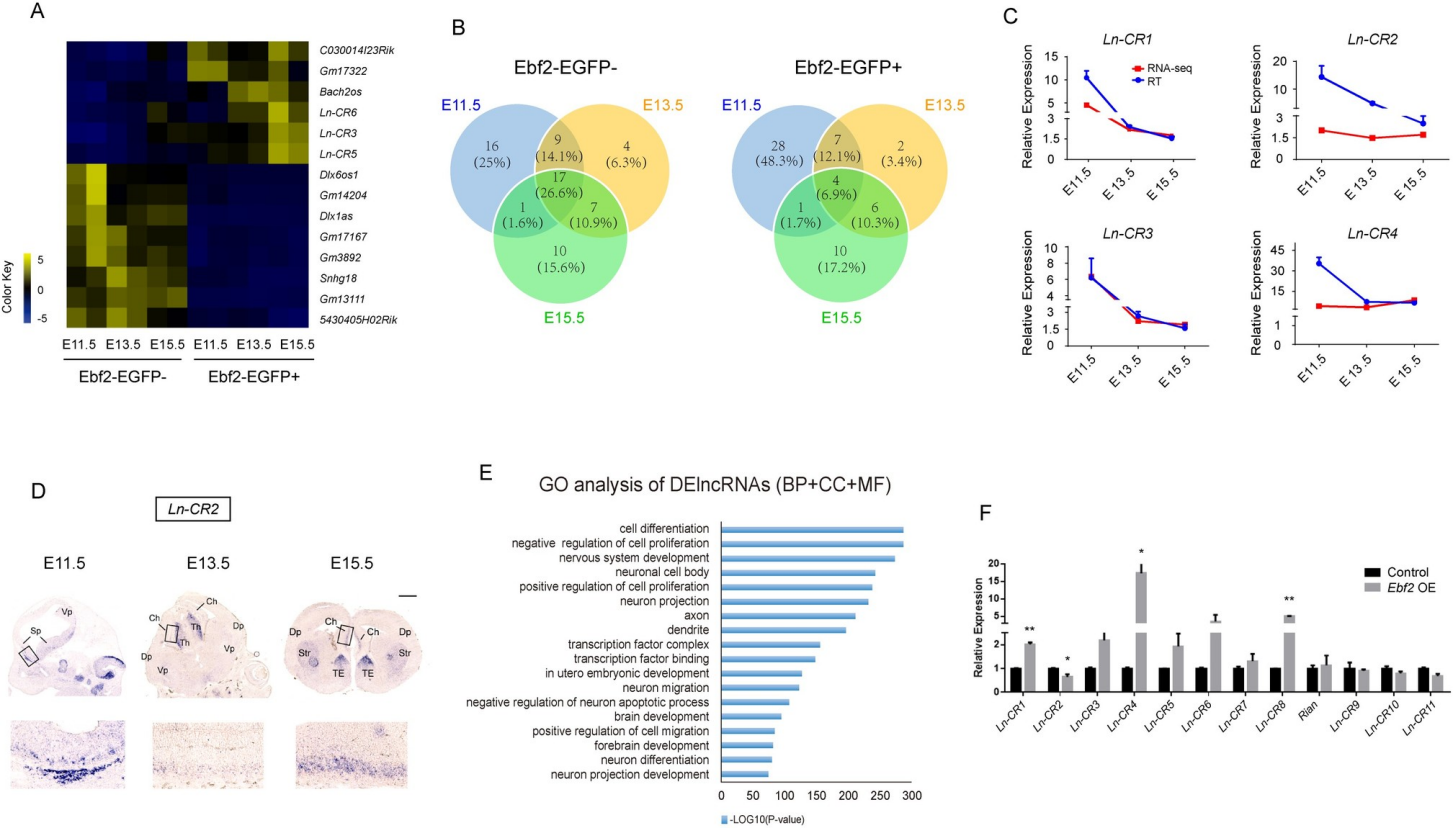
Characterization of differentially expressed lncRNAs in the Ebf2-EGFP+ and Ebf2-EGFP- cells. (A) Heatmap representing all differential expressed lncRNAs (FDR<0.05, p-value<0.05) in Ebf2-EGFP+ & Ebf2-EGFP- cell samples at the three embryonic stages. (B) Venn diagram displaying the extent of overlapping between DElncRNAs at the three embryonic stages. (C) The expression pattern of DElncRNAs between Ebf2-EGFP+ or Ebf2-EGFP- cells is consistent revealed by RNA-seq and qRT-PCR. Data represent mean ± SEM (n = 3 independent experiments). (D) *In situ* hybridization showing the endogenous expression of CR-specific lncRNA *Ln-CR2* at E11.5, E13.5 and E15.5, respectively. Regions with specific expression are labeled and higher magnification images of boxed areas are shown below. Abbreviations: Sp, septum; Vp, ventral pallium; Th, thalamus; Dp, dorsal pallium; Ch, cortical hem; Str, striatum; TE, thalamus eminence. Scale bar, 900 μm. (E) Enriched functional assessments (including biological processes, cellular components and molecular function) of highly expressed DElncRNAs (fold change ≥ 2) in Ebf2-EGFP+ or Ebf2-EGFP- cell population. (F) qRT-PCR analysis comparing the expression levels of lncRNAs in primary neural cell culture assay after virus infection with control (H1) or *Ebf2*-overexpression. Data represent mean ± SEM (n = 4 independent experiments, *P<0.05, Student’s T test).

From our RNA-seq analysis, we identified 2417 long non-coding RNA transcripts in cortical cells using the Cufflinks method. 200 lncRNAs were differentially expressed between the Ebf2-EGFP+ and the Ebf2-EGFP- population and termed DElncRNAs (FDR < 0.05, P-value < 0.01) (Fig 3A). 58 lncRNAs were highly expressed (2 folds higher) in Ebf2-EGFP+ cells compared to Ebf2-EGFP- cells, while 64 lncRNAs showed the opposite pattern. We further filtered DElncRNAs with these criteria: 1) specifically expressed in Ebf2-EGFP+ cells with FPKM at least 2-fold higher than that in Ebf2-EGFP- cells (Fig 3B); these lncRNAs also showed temporal specific expression patterns. 2) lncRNAs from 1) with FPKM ≤ 15 in Ebf2-EGFP- cells during all three stages, and FPKM ≥ 5 in Ebf2-EGFP+ cells at E15.5. We identified 9 lncRNAs that met the criteria and these had never been studied before in the context of neural development (S4 Table). We defined these lncRNAs as CR-specific lncRNAs and called *Ln-CR*# for simplicity. Next, the qPCR results to validate the lncRNAs mRNA expression level were generally in agreement with the RNA-seq data (Figs 3C and S3A). Notably, we detected strong expression of *Ln-CR2* in E11.5 Ebf2-EGFP+ cells, but comparably lower or even no signals in Ebf2-EGFP- cells, indicating Ebf2-EGFP+ cell-specific expression of *Ln-CR2* (Fig 3C and S4 Table). ISH on E11.5, E13.5 and E15.5 mouse embryonic brain sections revealed that *Ln-CR2* was selectively expressed in the known CR neuron origins such as Sp (septum), Ch (cortical hem) regions, as well as the TE (thalamus eminence), one of the extra-cortical origins of CR neurons, indicating cell-type specificity (Fig 3D).

Furthermore, we performed GO analysis (for more details, see S1 Text) of DElncRNAs to predict their potential functions (Fig 3E). We found significant enrichments of neuronal development process related GO terms, such as “nervous system development”, “neuron migration”, “negative regulation of neuron apoptotic process” and “neuron differentiation”, neuronal identities related GO terms such as “neuron projection”, “axon” and “dendrite”, as well as transcription factors regulation related GO terms such as “transcription factor complex”, “transcription factor binding”. The functional annotation of DElncRNAs identified from our sequencing data suggests that these lncRNAs are potential players in the processes of neurogenic lineage progression, neuronal maturation and transcription factor regulation (Fig 3E). Similar to CR-specific coding genes study, we also performed Ebf2-overexpression and Foxg1-knockdown experiments to predict lncRNAs’ potentials involved in CR neuron differentiation. Overexpression of *Ebf2* significantly up-regulated expression levels of CR-specific DElncRNAs *Ln-CR1*, *Ln-CR4* and *Ln-CR8* but not others such as *Ln-CR2* (Figs 1F and 3F). We also performed knockdown of *Foxg1*, which significantly up-regulated *Ln-CR3* and *Ln-CR5*. Some lncRNAs, for example, *Ln-CR1* also had the trends of up regulation, while others had not, such as *Ln-CR2* and *Ln-CR7*, implying that CR-specific lncRNAs might be involved in different regulatory pathways of CR neuron development (S3B Fig).

### Functional validation of CR-specific lncRNAs from *in vitro* and *in vivo*

**Fig 4:**
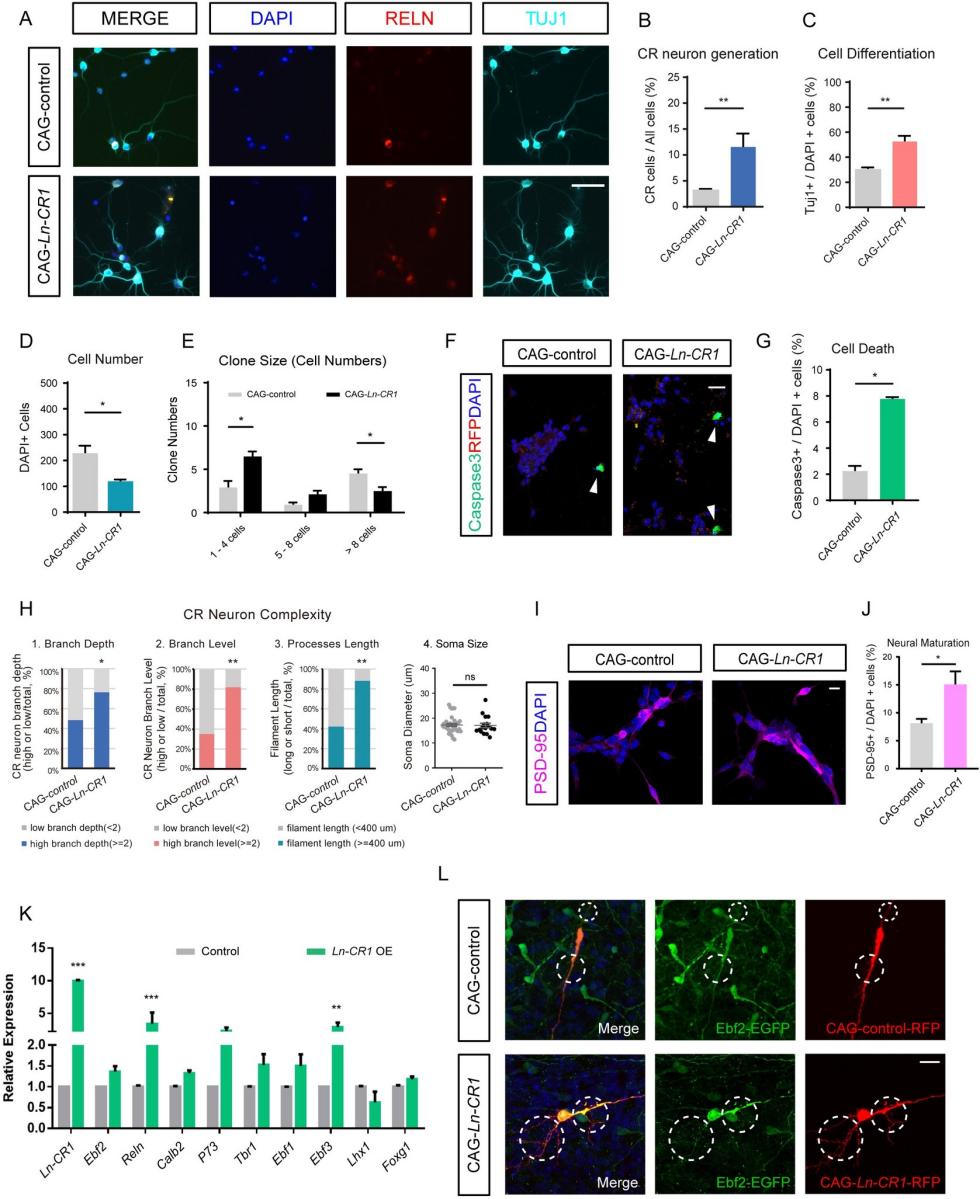
Functional validation of CR-specific lncRNAs *Ln-CR1 in vitro* and *in vivo*. (A) Immunostaining of neural cells treated with control (CAG-control) or overexpression lentiviruses for CR-specific lncRNA *Ln-CR1* (CAG-*Ln-CR1*) after *in vitro* culture for 5 days. RELN (red), TUJ1 (cyan), or DAPI (blue) for CR neurons, neurons, and nucleus, respectively. Scale bar, 50 μm. (B-G) Overexpression of CR-specific lncRNA *Ln-CR1* significantly increased CR neurons number, promoted NSC differentiation, generated more neuron-like small cell clones and also increased cell apoptosis. Caspase3 (green), DAPI (blue) for apoptotic cells (arrowheads) and nucleus, respectively. Scale bar, 20 μm. All the experiments were performed at least 3 times, and 6 randomly selected microscopy fields were counted each time. Data represented mean ± SEM. P values indicated were calculated by Student’s t test (unpaired). **p<0.01, *p<0.05. (H) Quantification of process branch depth, branch levels, total dendrites length and soma size of CR neurons after overexpression of CR-specific lncRNA *Ln-CR1* compared to control. All the experiments were performed 3 times, and 8 randomly selected microscopy fields were counted each time. Data represented mean ± SEM. p values indicated were calculated by Student’s t test (unpaired). **p<0.01, *p<0.05. (I-J) Immunostaining of neural cells treated with control (CAG-control) or overexpression lentiviruses for CR-specific lncRNA *Ln-CR1* (CAG-*Ln-CR1*) after *in vitro* culture for 5 days shown more mature neurons in *Ln-CR1* overexpression group. PSD-95 (magenta), DAPI (blue) for mature neurons and nucleus, respectively. Scale bar, 10 μm. The experiments were performed at least 3 times, and 6 randomly selected microscopy fields were counted each time. Data represented mean ± SEM. P values indicated were calculated by Student’s t test (unpaired). *p<0.05. (K) Quantification expression level of CR neuron molecular markers as *Reln*, *Ebf2*, and *Calb2* at 72h after lentiviral transduction in the NSC culture assay comparing the effects of overexpression of *Ln-CR1* to control. Data represented mean ± SEM (***p<0.001, **p<0.01, n>3, Student’s t test (unpaired)). (L) Comparison of dendritic spine, filopodia and processes in cortical wholemounts overexpressing *Ln-CR1* (CAG-*Ln-CR1*) to CAG-control. Scale bar, 20 μm. Ebf2-EGFP+ cortical wholemounts were collected from P3 pups after *in utero* electroporation to cerebral cortex layer 1 on E15.5.

In this study we have identified CR-specific lncRNAs, however the functional roles of these lncRNAs in neurogenesis have not been studied before. Using lentivirus-mediated overexpression, we performed *in vitro* and *in vivo* functional assays. We cultured single E11.5 cortical progenitor cells in adherent assays for 5 days (DIV5), then fixed the culture and stained for neuronal markers β-III tubulin (immature neurons) and *Reln* (CR neurons) (Figs 4A and S4A). In the control culture 29.89% of the cells were β-III tubulin+ neurons, of which 3.16% were Reln+ (Figs 4B and S4B). Overexpression of *Ln-CR1*, *Ln-CR2* and *Ln-CR3* increased the number of β-III tubulin+ cells and reduced total cell number (Figs 4C and 4D and S4C and S4D). Notably, Reln+ neurons were increased to 11.56% by *Ln-CR1* overexpression, 6.09% and 6.83% by *Ln-CR2* and *Ln-CR3* overexpression, respectively (Figs 4B and S4B). Interestingly, the number of small clones with less than 4 cells and all β-III tubulin+ cells increased in CAG-*Ln-CR1*, CAG-*Ln-CR2* and CAG-*Ln-CR3* compared to CAG-control, while the number of clones with more than 8 cells were much fewer than that in control group (Figs 4C and 4E; S4C–S4E). These results suggested that overexpression of CR-specific lncRNAs promoted neuronal differentiation. To investigate whether the loss in cell number is due to cell apoptosis, we stained the culture of control and *Ln-CR1* overexpression groups for Caspase-3 and found Caspase-3+ cells appeared significantly more in *Ln-CR*1 overexpression group. As the total number of β-III tubulin+ cells and Reln+ cells were actually increased, it is likely that overexpression of *Ln-CR1* promoted neural progenitor cell-specific cell death (Fig 4F and 4G).

During the postnatal stage, CR neurons developed complex morphology [55]. We also observed that overexpression of *Ln-CR1* altered the morphology of Reln+ neurons, increased whole processes length, branch depth and branch levels, while no significant differences were detected in neuron soma size (Figs 4A, 4H and S4F). To investigate whether overexpression of *Ln-CR1* affects neural maturation, we stained the cells for PSD-95, a synaptic marker, and found that PSD-95+ cells were significantly more in *Ln-CR1* overexpression group compared to that in the control group (Fig 4I and 4J). We further analyzed the effects of *Ln-CR1* on cell morphology in an *ex-vivo* culture system using the wholemount cortical hemispheres isolated from E15.5 Ebf2-EGFP+ embryos and cultured for 10 days (DIV10) to mimic late embryonic to postnatal development. Compared to control, we found that overexpression of *Ln-CR1* increased the morphological complexity of CR neurons, leading to filopodia, spine-like structures and bouton-like structures (S4G and S4H Fig). We observed similar results in P3.5 embryos in which *Ln-CR1* was overexpressed by *in utero* electroporation into the layer 1 of the cerebral cortex at E15.5 (Fig 4L).

To uncover how overexpression of these CR-specific lncRNAs molecularly affected CR neuron development, we assessed the mRNA levels of several known CR neuron-related genes by qPCR (Figs 4K and S4I). We found genes highly expressed in CR neurons such as *Reln*, *Ebf2*, *Calb2* and *Trp73*, *Ebf1*, *Ebf3*, *Tbr1*, *Nhlh2* and *Lhx1*, were selectively altered when overexpressing CR-specific lncRNAs candidates *Ln-CR1*, *Ln-CR2* and *Ln-CR3*. The fact that typical CR markers expressed in major CR neuron populations such as *Reln*, *Ebf3*, *Ebf2* and *Trp73* were significantly upregulated under one particular lncRNA overexpression (Figs 4K and S4I), implying that these lncRNAs might have great potentials in regulating the molecular pathways for CR neuron differentiation.

### Single-cell analysis identifies heterogeneities within a pure CR neuron population

**Fig 5:**
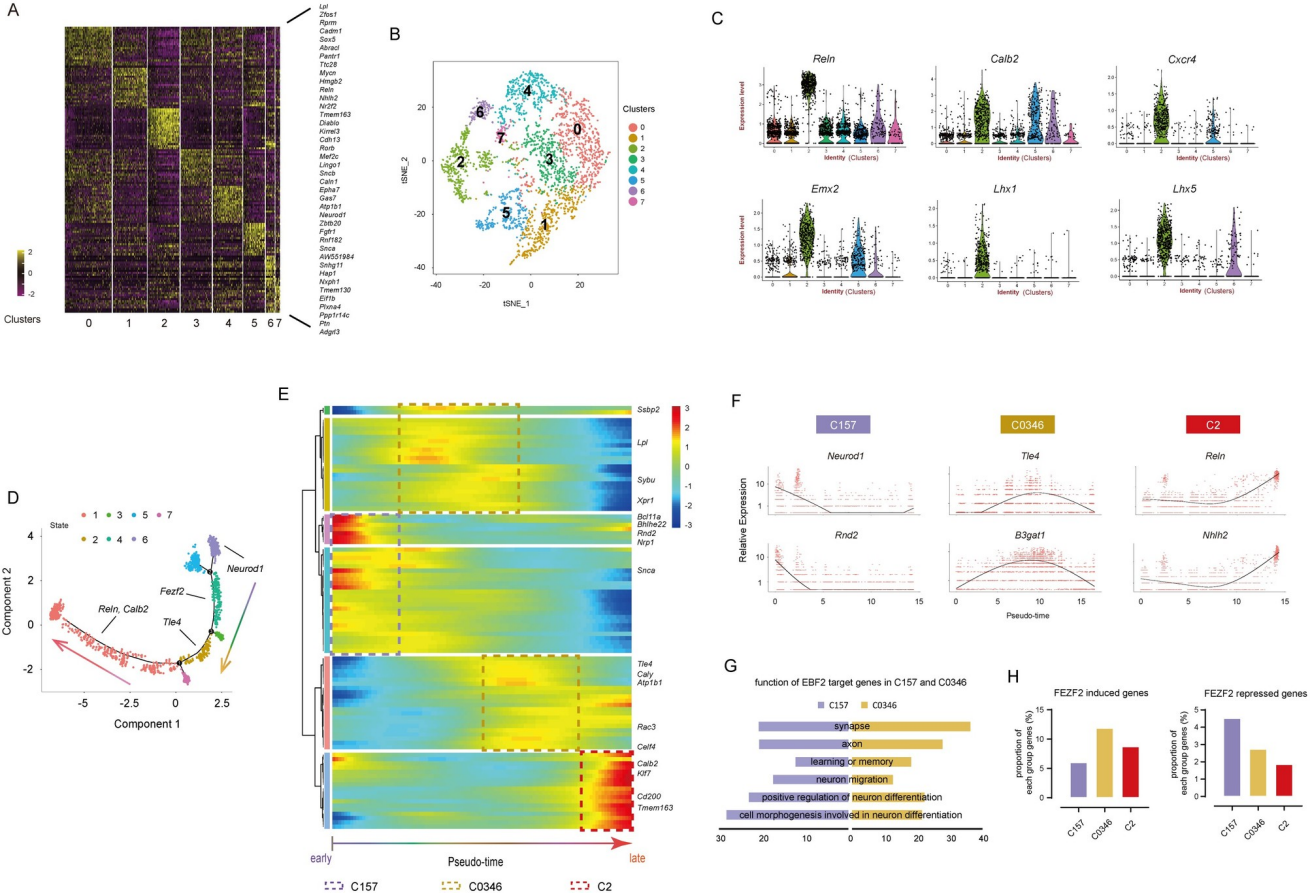
Single cell RNA-seq identify heterogeneities within CR neuron population and reveal genetic program for early neuron differentiation. (A) Heatmap of differential expressed genes in E15.5 Ebf2-EGFP+ cells subpopulations. (B) The K-means algorithm-based plot showing eight major subpopulations. (C) Violin plots showing genes expressed in CR neuron origins with high and specific expression in some cell subpopulations. Each dot represents a single cell. (D) Pseudotime trajectories of Ebf2-EGFP+ cells computing reconstructed by Monocle. Cell states are indicated by colors and sequential numbers are in the black circles. (E) Heatmap shows gene dynamics along pseudotemporal cortical neuron differentiation. X-axis shows pseudotime ordering from early to late, Y-axis shows a single gene expression level dynamic per row. Gene expression level from high to low is indicated by colors from red to blue. (F) Expression level dynamics across pseudotime ordering of C157 genes *Neurod1*, *Rnd2*, C0346 genes *Tle4*, *B3gat1*, and C2 genes *Reln*, *Nhlh2* are shown. (G) Functional GO analysis for EBF2 target genes in C157 and C0346, respectively. (H) The distribution of FEZF2 targeting induced genes and repressed genes across C157, C0346 and C2.

We have previously shown that in the Ebf2-EGFP transgenic mice, EGFP is specifically expressed in layer 1 neurons and more than 80% of dissociated E15.5 Ebf2-EGFP+ cells are Reln+ [19]. In the cortical brain sections of E15.5 Ebf2-EGFP mice, 98.67% were Reln+, indicating that Ebf2-EGFP+ cells consist of a nearly pure population of Reln+ neurons (S3D Fig). The Ebf2-expressing cells consist of CR neurons from different origins including cortical hem, septum and ventral pallium [22,23,55]. To investigate the molecular heterogeneity and characterize the developmental states of CR neurons at single cell level, we performed single-cell RNA-seq (10X genomic Chromium) of 3000 Ebf2-EGFP+ cells purified by FACS of E15.5 cortical cells. After filtering out potential noises as multiplet cells and mitochondria genes, then normalization and PCA analysis, the acquired gene expression data sets were selected for the following analysis (S5A–S5C Fig). Through K-Means clustering, Ebf2-EGFP+ cells at this stage were molecularly clustered into eight major subpopulations, well separated and visualized in the t-SNE plot (Fig 5A and 5B). These eight cell clusters had distinct gene expression patterns and were distinguished by highly differential expressed gene markers (Figs 5A and S5D). Notably, Cluster 2 was the most segregated group from the others, with high expression level of typical CR markers such as *Reln* (Fig 5A). Interestingly, we found that 46% of E13.5 CR genes and 50.6% of P2 CR genes identified from a previous study [23] were included in Cluster 2 (29 genes, and 44 genes, respectively), indicating high enrichments of common CR genes in this cluster.

CR neurons were reported to be generated from many areas of developing pallium and redistributed across the cortical surface via tangential migration [23,41,56,57]. It is still elusive whether CR neurons arising from different origins are molecularly distinguishable and maintain their different original identities, or develop to the same type of cells with identical gene expression profile after they reach the cortical layer 1. We investigated whether the eight cell clusters expressed different regional genes. We found that typical CR marker genes such as *Reln* and *Calb2* were detected in all cell subpopulations. Surprisingly, we did not detect segregation of region-specific genes in the differential expressed genes (DEGs) of eight cell clusters. In contrast, we observed genes expressed in various CR cell original territories were enriched in the same cell cluster. For example, *Cxcr4* (cortical hem), *Emx2* (VZ), *Lhx1* (septum, ventral pallium), *Lhx5* (cortical hem, septum and ventral pallium) [41,56,57] were all included in Cluster 2 (Figs 5C and S5E), suggesting single cells expressed genes of different positional origins. Therefore, our results showed that different CR neuron origins cannot be segregated by unsupervised molecular clustering, implicating the possibility that CR neurons may lose the original region identities once they migrate into the cerebral cortex layer 1, and molecular and functional maturation of CR neurons may follow a general path regardless of their origins.

### Reconstruction of CR neuron developmental lineage reveals a transition from activation of a common neuronal differentiation program to repression of other neuronal subtypes to establish the CR cell-specific identity

To characterize the developmental insights of the CR neurons, we performed pseudotime reconstruction using Monocle analysis [58]. We chose genes that were expressed across more than 10 cells and their average expression levels were more than 1 for this analysis (Figs 5D and S5F). We found that the eight major cell subpopulations clustered from t-SNE plot corresponded to the temporal cortical neuron development progression from early to late stages (Fig 5E). Genes in Cluster 5 could be the first activated population during the pseudo-temporal ordering, sequentially followed by Cluster 1 and 7 genes, which displayed high expression levels at the beginning of the time period, dropped to low levels later. For example, Cluster 5 gene *Neurod1* is known to be expressed in committed neuronal progenitor cells in the upper SVZ and in newborn neurons at the lower IZ and plays a prominent role in cell fate specification [59]. Cluster 1 gene *Rnd2* functions in the downstream of *Neurog2* to promote neuron migration [46]. Other genes in these three cell clusters such as *Plxna4* is also reported to regulate neuronal migration in neocortex development (Fig 5E and 5F) [47]. Thus, Cluster 1, 5, 7 (C157 in short) genes might be involved in initial transition from progenitor cells to neuronal differentiation. In contrast, genes in Cluster 2 were expressed at lower expression levels at the starting site, but higher expression levels at the end. The top differentially expressed genes in this cluster were CR neuron markers, such as *Reln*, *Trp73*, *Calb2*, *Ebf2*, *Lhx5*, implying a more committed CR neuron state (Figs 5E and 5F and S5G). Cluster 0, 3, 4, 6 (C0346) genes were in an intermediate state, displaying higher expression levels during the middle period of the pseudo-temporal axis than the start and terminal points. Other layer-specific neuron markers fell into C0346, showing transient expression in Ebf2-EGFP+ cells. For example, the deep layer neuron marker *Fezf2* and *Tle4* were enriched in Cluster 3 and 4 respectively (Figs 5E and 5F and S5G). Therefore, our pseudo-timing analysis implies that differentiation of CR neurons starts first from a general neuronal differentiation program, then goes through a refinement of gene expression to suppress other cell type-specific genes, and finally reaches an establishment of the CR neuron-specific genes.

To investigate whether the temporal dynamics in gene expression derived from single cell sequencing analysis are consistent with the *in vivo* developmental progression, we compared the gene expression profile of the three pseudo-reconstructed subpopulations (C157, C0346, C2) with the temporal pattern identified at the three developmental stages (E11.5-E13.5-E15.5) (Figs 2G and 5E and 5F). To better represent each cluster’s specific gene expression pattern, we focused on the top 20% of the highly expressed genes of each cell cluster. Interestingly, we found that 38.6% (64/166) of the “up-up-up” class genes were C2 genes, including reported CR genes (e.g. *Reln*, *Trp73*, *Lhx5*, *Calb2*, *Ebf2*, *Ebf3*, *Lhx1*, *Cdkn1a*), CR-specific genes identified in this study (e.g. *Nhlh2*, *Ppp2r2c*), and other genes related to mature neurons (*Kcnip2*, *Nxph3*, *Tmem163*) (Figs 2G, 5E, 5F and S5G and S1 and S3 Tables). *Neurod1*, *Rnd2* and *Plxna4*, genes appeared in the early state C157 cluster were found in the “up-unchange-down” and “up-down-down” classes (Fig 1E and 1F and S3 Table), in line with their trends with higher expression at the initial stage and lower expression later. Genes in the “unchange-unchange-up” and “unchange-up-up” classes genes tended to be in the intermediate state C0346, such as *Tle4*, *Fezf2* (Figs 5E and 5F and S5G and S3 Table). Hence, both transcriptome analysis at single and bulk cell level identify a transcriptional cascade from general neuronal differentiation to specification of a neuronal subtype.

### Transcriptional factors involved in transitions in gene expression during constraining terminal cell fates to CR neurons

To understand the molecular mechanisms underlying the transition from general neuronal fate to committed CR neurons identities, we focused on the genetic network of key transcription factors that may predominate the differentiation progression. *Ebf2* is enriched in Cluster 2 and its target genes has been identified in Neurog2-induced neurogenesis assays recently [60]. We found that 457 of the early phase C157 genes and 611 of the intermediate C0346 genes were EBF2 target genes during neural differentiation, implying that it might affect the molecular pathways of CR neuron differentiation (S5 Table). Through GO analysis, we found the target genes in C157 were involved in neuron differentiation and neuron migration, while the target genes in C0346 were more correlated with synapse, axon, learning and memory properties (Fig 5G). Similarly, 132 genes in C157 and 172 genes in C0346 were the target genes of CR marker ZIC2 (Cluster 2) (S5H Fig and S5 Table) [61]. Therefore, it raised a possibility that these genes might interact and regulate non-CR genes to allow CR-specific traits to be established. On the other hand, *Foxg1*, a gene known to suppress CR cell fate [28,62], is highly expressed in C157 and C0346 but low in C2 (S5I Fig). From our results and former studies, knockdown of *Foxg1* also significantly increased C2 genes expression like *Reln*, *Ebf2*, *Nhlh2* and promoted CR neuron generation from *in vitro* and *in vivo* assays (S1F Fig) [28,29,62], while *Ebf2* suppressed the expression level of *Foxg1* (S1E Fig). This implies that suppression of C157 and C0346 genes facilitates the expression of C2 genes. Interestingly, *Fezf2* is an intermediate state C0346 gene. It is known to encode a key transcription factor for deep layer neurons. *Fezf2* regulates the expression of gene sets that are responsible for establishing mouse corticospinal motor neurons identities [63]. Here, we found that FEZF2-induced genes were more frequently appearing in C2 than C157, while FEZF2-repressed genes were less in C2 than in C157 (Fig 5H and S5 Table), suggesting the C0346 gene *Fezf2* might induce CR neuron identities via activation of C2 genes and inhibition of the general cortical neuron fate by repressing C157 genes. Taken together, a subset of key transcription factors might play a pivotal role in controlling distinct differentiation events through combinatorial actions on a variety of temporal genetic targets and networks, thus guiding initial generic cortical neuron program to terminal CR neuron-specific cell fate determination.

### Distinct H3K27ac and H3K27me3 histone modification patterns in Ebf2-EGFP+ cells

**Fig 6:**
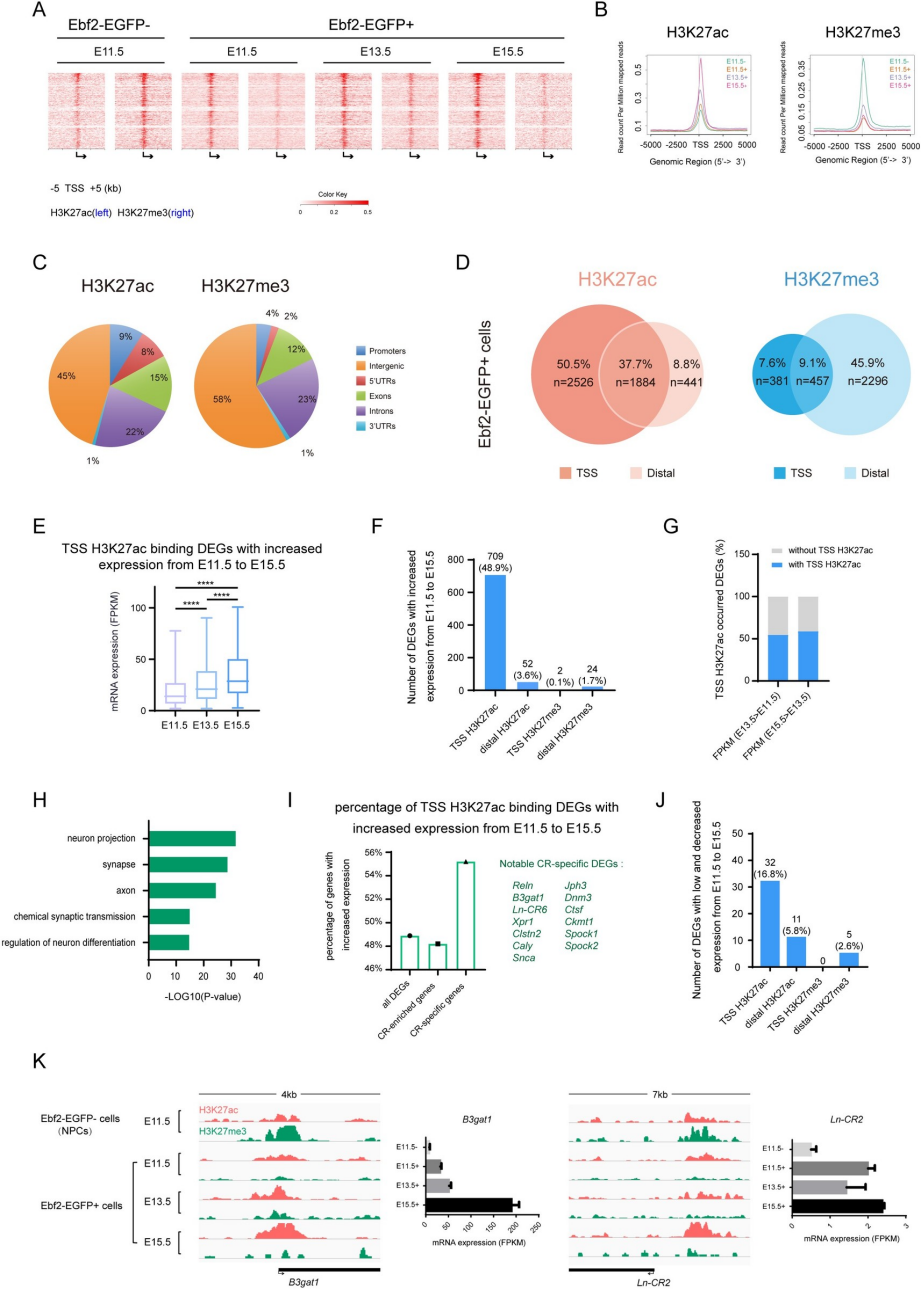
Genomic features of H3K27ac and H3K27me3 histone modification in the early differentiating neurons. (A) Heatmap representation of ChIP-seq signal density for H3K27ac and H3K27me3 for 10 kb centered on predicted gene TSSs in Ebf2-EGFP- cells (E11.5) and Ebf2-EGFP+ cells (E11.5, E13.5 and E15.5), respectively. (B) A profile plot showing normalized H3K27ac (left) or H3K27me3 (right) enrichments around TSS of all detected genes in Ebf2-EGFP- cells (E11.5) and Ebf2-EGFP+ cells (E11.5, E13.5 and E15.5), respectively. (C) Genomic features of H3K27ac and H3K27me3-bound genes in Ebf2-EGFP+ cells. (D) Proportion of TSS and distal H3K27ac, H3K27me3 bindings distributed in DEGs in Ebf2-EGFP+ cells, respectively. (E) mRNA expression (FPKM) of DEGs with H3K27ac signals around TSS region from E11.5 to E15.5. Data represent mean ± SEM, N = 334 genes, independent experiments, ****P<0.0001, T-test. (F) The number of DEGs that steadily increase expression level from E11.5 to E15.5 with TSS and distal region H3K27ac, H3K27me3 occupancies in Ebf2-EGFP+ cells. The percentage indicate the proportion of all E11.5 to E15.5 expression increased DEGs (with and without histone modification). Chi-square test showing the significant correlation between H3K27ac signals in the gene TSS regions and gene expression, P<0.01. (G) Percentage of DEGs that steadily increase expression level from E11.5 to E13.5, E13.5 to E15.5, with (blue) or without (grey) TSS H3K27ac enrichments, respectively, in the Ebf2-EGFP+ cell populations, that is, during CR neuron differentiation progression. (H) Functional GO analysis for those TSS H3K27ac binding DEGs in Ebf2-EGFP+ cells with steadily increased expression level from E11.5 to E15.5. Genes for analysis derived from Fig 6G. (I) Percentage of gene number (DEGs, CR-enriched DEGs and CR-specific DEGs) with positive correlation between expression (FPKM) and H3K27ac signals in the gene TSS region. Notable CR-specific genes are listed. (J) The number of DEGs that lowly expressed (FPKM<1) and steadily decreased expression level from E11.5 to E15.5 with TSS and distal region H3K27ac, H3K27me3 occupancies in Ebf2-EGFP+ cells. The percentage indicate the proportion of all E11.5 to E15.5 low and decreased expression DEGs (with and without histone modification). (K) The TSS regions of CR-specific genes *B3gat1*, and CR-specific lncRNAs *Ln-CR2* demonstrate a similar pattern of histone modification during early embryonic development (bivalent in NPCs and become more activated with monovalent H3K27ac in Ebf2-EGFP+ cells from E11.5 to E15.5).

Histone modification is a critical determinant of transcriptionally active or silent chromatin states [64]. To gain a comprehensive view of cell type-specific histone modification in early neural progenitor cells and differentiating neurons, we performed H3K27ac and H3K27me3 chromatin immunoprecipitation followed by high-throughput sequencing (ChIP-seq) in Ebf2-EGFP+ cells at early cortical developmental stages (E11.5, E13.5, E15.5) and Ebf2-EGFP- cells at E11.5 (mostly NSCs and neural progenitor cells at this stage). We analyzed the genome wide and gene-specific occupancy of these histone modifications in combination with the RNA-seq results on these cell types. First, our data showed strong concordance in H3K27ac and H3K27me3 bindings across two biological replicates (S6A and S6B Fig). According to a general strategy used to classify regulatory elements (REs) [65,66], we classified H3K27ac and H3K27me3 bound TSS (± 5kb) by the pattern of histone modification as TSS activation (monovalent H3K27ac), repression (monovalent H3K27me3) and poised (bivalent H3K27ac and H3K27me3). We observed bivalent H3K27ac and H3K27me3 bindings at 58.3% of all detected genes TSS sites in Ebf2-EGFP- cells (monovalent H3K27ac bindings at only 0.9% of the genes and monovalent H3K27me3 bindings at only 4.5% of the genes). In contrast, bivalent H3K27ac and H3K27me3 bindings at only 16.4% of the TSS sites of all genes in Ebf2-EGFP+ cells, while monovalent H3K27ac bindings occurred in the majority of genes (72.4%), monovalent H3K27me3 bindings occurred in only 0.5% of the genes (Fig 6A and 6B). Such observations were consistent with previous studies that bivalent histone modification patterns formed a poised state to be ready for initiation of further developmental events in the ES cells or progenitor cells, while monovalent states presented in the differentiated cells indicated more organized states in the established specific cell identity [66,67]. Compared to E11.5 Ebf2-EGFP- progenitor cells, in which H3K27ac enrichments at TSS were the weakest while H3K27me3 were the strongest among the tested groups, in E11.5 Ebf2-EGFP+ cells, H3K27ac enrichments at TSS were stronger and became increasingly stronger while H3K27me3 bindings remain steady at a weaker level as development proceeded from E11.5 to E15.5 (Fig 6A and 6B). These patterns suggest that histone modification patterns are different between early differentiating neurons and neural progenitor cells, and dynamically change as early-born neurons differentiate.

We further analyzed the genomic location of H3K27ac and H3K27me3 bindings in Ebf2-EGFP+ cells (Figs 6C and S6C–S6H). We found that the majority of H3K27ac and H3K27me3 binding peaks were located in the intergenic and intron regions, while less than 10% of the peaks were distributed in the promoter regions (Figs 6C and S6C and S6D). At E11.5, the genomic distribution patterns of H3K27ac and H3K27me3 in Ebf2-EGFP- cells were similar to that in Ebf2-EGFP+ cells (S6E–S6H Fig).

Interestingly, a distinct histone modification pattern emerged when we focused on the TSS regions and the distal regions (within 100kb) of DEGs. TSS and distal regions were reported to have important regulatory potentials in the development system [68,69], so we concentrated on these two regions of DEGs in Ebf2-EGFP+ cells. We found that for the H3K27ac bindings, 88.2% (4410) of the genes had peaks around TSS, 46.5% (2325) had peaks around distal region. The majority of the genes either had H3K27ac peaks on only TSS (50.5%, 2526 genes) or on both TSS and distal regions (37.7%, 1884 genes), while much fewer genes had peaks on only distal regions (8.8%, 441 genes) (Fig 6D). Conversely, for the H3K27me3 modification, 55.0% (2753) of the genes had bindings on distal regions, only 16.7% (838) of the genes had bindings on TSS. The majority of genes had H3K27me3 peaks on only distal regions (45.9%, 2296 genes), while a small portion of genes showed peaks in both TSS and distal regions (9.1%, 457 genes), or only TSS (7.6%, 381 genes) (Fig 6D). These comparisons revealed that more genes occupied with H3K27me3 enrichments on distal region than on TSS, which is quite different from the H3K27ac pattern. Hence, DEGs might be activated in the Ebf2-EGFP+ cells as the most of the genes (88.2%) were occupied with H3K27ac enrichments, for example, CR marker genes *Calb2*, *Trp73* and *Tbr1*, and novel CR-specific coding genes and lncRNAs identified in this study, especially *Sncb*, *Ppp2r2c*, *Jph3*, *Ln-CR2* and *Ln-CR3*. The histone modification pattern of DEGs with H3K27ac at TSS region and H3K27me3 at distal region in Ebf2-EGFP+ cells might contribute to maintenance and promotion of CR neuron differentiation.

### Differentiation genes are facilitated during CR neuron specification accompanied by TSS H3K27ac bindings

It has been shown that chromatin state around TSS or distal region is a strong indicator of RE function [67,68]. We noticed that among 1555 CR-enriched genes, 97.1% of them were occupied by H3K27ac bindings (S6I Fig). Distal H3K27ac bindings are known to activate gene expression in many development systems including the nervous system [68,69]. Interestingly, we found that 46.5% (2325) of DEGs had distal H3K27ac bindings in Ebf2-EGFP+ cells (Fig 6D), however only 52 genes showed increased expression level from E11.5 to E15.5 (Fig 6E and 6F). In contrast, we found that genes with H3K27ac bindings surrounding TSS regions tended to have increasing expression levels as development proceeds. Genomically, 88.2% (4410) of DEGs carried TSS H3K27ac bindings in Ebf2-EGFP+ cells (Fig 6D), 45.7% (2288) of DEGs had consistent TSS H3K27ac modification across the three stages, and 31.0% (709) of them showed stably increased expression from E11.5 to E15.5 (48.9% of all increased DEGs), suggesting H3K27ac modification around TSS significantly increased temporal genes expression (Chi-squared test: p-value < 0.01) (Fig 6E and 6F). These genes significantly encoded early differentiating neuron characters and functions, like “neuron projection”, “synapse”, “axon”, “chemical synaptic transmission”, “regulation of neuron differentiation” (Fig 6G and 6H). Genes accompanied by TSS H3K27ac bindings across the three developmental time points also occupied 75% (93) of the highly expressed C2 genes (top 20%) from single cell pseudotime lineages (S6J Fig), implicating their constraints to CR neuron termination. For CR-enriched DEGs, 340 with TSS H3K27ac modifications showed steadily increased expression along the time period (48.2% of all increased CR-enriched DEGs). For CR-specific DEGs, 32 were such genes (55.2% of all increased CR-specific DEGs), including *Reln*, *B3gat1*, *Ln-CR6*, *Xpr1*, *Clstn2*, *Caly*, *Snca*, *Jph3*, *Dnm3*, *Ctsf*, *Ckmt1*, *Spock1*, *Spock2* (Fig 6I). We wondered whether TSS H3K27ac also contributed to repression of gene expression, however, only 1.4% (32) of such histone modified DEGs showed low (FPKM<1) and decreased expression from E11.5 to E15.5 (16.8% of all low and decreased expressed DEGs) (Fig 6J). Hence, promoter but not distal region H3K27ac modifications contribute to up-regulated gene expression, especially CR-specific genes along differentiation. We noticed that 16.7% (838) and 55% (2753) of DEGs had TSS and distal H3K27me3 bindings in Ebf2-EGFP+ cells, respectively (Fig 6D), while only 2 and 24 genes showed increased expression over the three stages (Fig 6F). On the other hand, distal H3K27me3 was not associated with suppressing gene expression because only 11 low expressed genes carried this type of modification (S6K Fig). Therefore, the data showed that H3K27ac activation at promoter but not distal region in Ebf2-EGFP+ cells facilitated early neuron differentiation. Examples reflecting involvements of H3K27ac and H3K27me3 modifications in the differential expression level of CR-specific coding genes and lncRNAs included *B3gat1*, *Ln-CR2*, *Sncb* and *Ln-CR5* (Figs 6K and S6L). Their expression levels in Ebf2-EGFP+ cells increased from E11.5 to E15.5, accompanied by increasingly stronger H3K27ac enrichments at TSS regions during these stages. Meanwhile, these genes are expressed at low levels in Ebf2-EGFP- cells on E11.5, corresponding to H3K27me3 enrichments at TSS of these genes in Ebf2-EGFP- cells (Figs 6K and S6L). *Ln-CR2*, for example, which is increased in Ebf2-EGFP+ cells from E13.5 to E15.5, exhibits a change in histone modification with low H3K27ac peaks on the TSS region on E13.5 but high H3K27ac peaks on E11.5 and E15.5 (Fig 6K).

## Discussion

In this study, we characterized a temporal dynamic transcriptome profile of early neurogenesis and the gene expression signatures associated with preplate neuronal differentiation and subsequent establishment of CR cell properties. Furthermore, we discovered unique histone modification patterns which particularly emphasized on promoter regions reinforcing CR neuron specification. We also identified three distinct development states within a pure CR neuron population and uncovered molecular hallmarks along CR neuron differentiation, which had never been reported before. Our study unraveled the remarkable temporal dynamics in gene expression during differentiation of a specific neuronal subtype and provided molecular markers to extend the repertoire of preplate and CR neuron markers. Our findings provided a comprehensive molecular depiction of the early neuronal differentiation events, shedding new light on the mechanism underlying preplate differentiation and CR neuron specification, the first step of cortical neurogenesis.

### Molecular transition from preplate to CR neurons

As cortical neurogenesis proceeds, the preplate is physically separated into the subplate and the layer 1 by migrating cortical plate neurons. We first identified over 1044 coding genes and 40 lncRNAs with higher expression in the newly differentiated preplate neurons (Ebf2-EGFP+ cells) at E11.5 (Figs 1D and 3B and S1 and S4 Tables). At E13.5, 370 genes continue to show enrichments in preplate neurons, while additional 20 genes also have higher expression levels in subplate neurons. A subset of these genes continue to be differentially expressed in the Ebf2-EGFP+ cells at E15.5, while the total DEGs at E15.5 are much less than earlier stages, suggesting further specification of the CR cell fate. Indeed, we found fewer overlaps between the confirmed subplate neuron-specific genes at this age (28 genes overlap, 2.3% of total DEGs at E15.5) [18], confirming the molecular segregation of preplate neurons into the subplate and layer 1 by E15.5. The fact that the number of DEGs is the highest at E11.5 reflects the degree of the difference between the two populations: at E11.5, all Ebf2-EGFP+ cells are early differentiating neurons, while the Ebf2-EGFP- cells are mostly cortical progenitor cells. As the Ebf2-negative population includes both cortical plate neurons and neural progenitor cells at E13.5 and E15.5, genes common to neurons might not have significant changes. This dynamic transcriptional profile may underlie the progression of preplate differentiation to the acquisition of unique molecular identity of CR neurons and subplate. For example, 166 genes are enriched in E11.5 and highly expressed in preplate, then later become restricted in CR neurons, while 851 genes which are also enriched in E11.5 but no longer specifically expressed in layer 1 at E15.5.

Our transcriptome analysis also reveals a surprising enrichment of genes categorized in the GO terms (Fig 2B–2D and S2 Table) such as “neuron projection”, “neuron differentiation”, “axonogenesis”, even “synapse” and “synaptic transmission” as early as E11.5. Terms such as “gate channel activity” and “ion channel activity” also appear before E15.5. Interestingly, whole-cell patch-clamp recording showed an inward sodium current in putative CR cells as early as E12 [36]. In addition, dynamic expression of K+ channels and Ca2+ channels were also described in pre- and postnatal CR cells [12,36,70,71]. We found that Ebf2-EGFP-positive cells at E11.5 already expressed neurotransmitter receptors including ionotropic and metabotropic glutamate receptors, AMPA glutamate receptors, at E11.5 (S2 Table). Interestingly, it has been reported that at very early stages of neural development, subplate neurons form transient synapses with axons coming from the subcortical regions to guide later formation of cortico-subcortical connectivity [72,73]. Our results suggest that populations of early differentiating neurons, including Cajal-Retzius cells, are equipped with machineries suited for electric or chemical synaptic connections shortly after they are born. It’s possible that functional connections are established earlier than currently recognized. Alternatively, expression of these synapse-related proteins may not necessarily confer electrophysiological properties, but rather prime early differentiating neurons to be ready for synaptic connection once they encounter other cortical plate neurons.

GENCODE annotation suggests that nearly 40% of the differential expressed lncRNAs are brain system specific [74]. Emerging evidence has uncovered the crucial functions of lncRNAs relating to brain development, and defects of lncRNAs have also been revealed to cause severe human neural diseases such as Alzheimer’s disease and schizophrenia [75,76]. As the functions of lncRNAs are often cell type-specific, we are interested in identifying lncRNAs enriched in CR neurons and explore their functions. In this study, we discovered novel lncRNAs enriched in Ebf2-EGFP+ CR cells and selectively expressed in these CR neurons. We observed increased expression of these lncRNAs corresponding to Ebf2 overexpression or Foxg1 knockdown, implicating they might be regulated by these transcription factors during early corticogenesis. We also tested the potential functions of lncRNA candidates during CR cell differentiation, which establishes basis for further in-depth study.

Recent studies have shown that disease-related genes are highly expressed during early differentiation period, for example, *Nrxn1 is* enriched in subplate neurons and it is an autism-associated gene [18]. In our study, *Ln-CR1* locates within the first exon of *Klf7* which is a member of the Kruppel-like factor family. *Klf7* is highly expressed across the surface of the cerebral cortex as early as E11.5 [31]. It functions in regulating neuronal differentiation and promoting neurite outgrowth [77,78]. Our results show that *Ln-CR1* promotes neuronal differentiation and maturation, which is similar to *Klf7*’s function (Fig 4). As *Klf7* with a 2q33.3q34 deletion has been found to be a candidate gene for Autism, and the patients with this deletion also have microcephaly feature [79], it is possible that *Ln-CR1* could be also involved. In addition, overexpression of *Ln-CR1* greatly increase expression of *Ebf3* (Fig 4K), a gene reported to be associated with Intellectual Disability [80]. These findings suggest that CR-specific genes could be involved in the etiology of neurodevelopmental diseases.

### Single-cell RNA-seq analysis reveals distinct developmental states within pure CR neuron population

Pseudo-time analysis of single cell RNA-seq data has offered a way to further dissect the transitional difference in gene expression within a heterogeneous population. Here, we reported that by pseudo-time analysis, CR neurons could be classified into 3 subgroups. The first group consisted of activated cluster 1, 5 and 7 genes, which were highly expressed at the beginning of pseudo-temporal axis. We found that genes in this group were associated with characteristics of cells located in the upper VZ and lower cortical plate such as *NeuroD1*, representing transitioning from neural progenitor cells to newborn neurons [59,81]. The second group was enriched with cluster 0, 3, 4, 6 genes that were activated during the middle of pseudotime trajectory. These genes were categorized under “neural differentiation” “neurite outgrowth” and “migration” GO terms using DAVID analysis. The third group along the pseudo-time line consisted of mainly cluster 2 genes, which included many CR marker genes, synapse and axonogenesis-related genes. Interestingly, we found that genes first expressed in neural progenitor cells then in cortical plate neurons, such as *Neurod1* and *Rnd2*, were expressed higher first, then down-regulated in the third group, suggesting when cells undergo fate decisions to become CR neurons, genes related to other neuronal identities would need to be suppressed at the later differentiation stage. Indeed, transcription factors, such as EBF2 and ZIC2, expression levels of which increase as CR neurons differentiate, and FEZF2, which is highly expressed in the intermediate state, could form dynamic regulatory cascades via corporately repressing non-CR genes and inducing CR-specific genes to promote the fine molecular transition from a new-born neuron to a fully committed subtype. Further studies will help resolve the biological relevances of these analysis-acquired developmental trajectory of gene expression.

Overall, our study provides a comprehensive profile of gene expression for the early-born neurons in the cerebral cortex at both single cell and population level, unraveling the molecular dynamics during early neurogenesis.

## Methods

### Ethics statement

All animal protocols used in this study were approved by the Institutional Animal Care and Use Committee (IACUC) of Tsinghua University and performed in accordance with guidelines of the IACUC. The laboratory animal facility has been accredited by Association for Assessment and Accreditation of Laboratory Animal Care International (AAALAC).

### Mice

Transgenic mouse line Tg(Ebf2-EGFP)58Gsat/Mmcd in this study was created by the GENSAT Project [20], and was obtained from the NIH Mutant Mouse Regional Resource Center. The colony of this mouse line was generated in the animal facility of Tsinghua University. For timed mice mating, the day of the vaginal plug was considered E0.5.

### Dissociation and purification of CR cells and neural progenitor cells

Ebf2-EGFP positive and negative cells at E11.5, E13.5, E15.5 were dissociated, resuspended and purified by the fluorescent-activated cell sorting (FACS). Experimental procedures were as previously described [82]. Briefly, embryonic brains were dissected and transferred to HIB solution (30 mM KCl, 5 mM NaOH, 5 mM NaH2PO4 anhydrous, 0.5 mM MgCl2 × 6 H2O, 20 mM Na Pyruvate, 5.5 mM Glucose, 200 mM Sorbitol), and kept in the solution during dissection procedures. Cortical regions were dissected and tissues were incubated at 37°C for 30 min in papain solution containing DMEM medium (Life Tech), Na-pyruvate (Sigma), L-glutamine (Life Tech), NAC (Amresco), papain (Worthington). Tissues were triturated 5–10 times by 1ml pipette during papain treatment. After papain treatment, tissues were dissociated into single cell, washed three times by DMEM and centrifuged 405g for 10mins. The cell pellet was resuspended in HIB solution.

### FACS sorting

After dissociation, the Ebf2-EGFP positive and negative cells were collected by BD FACS inFlux v7 Cell Sorter using 100 μm nozzle diameter. The purity of FACS sorted Ebf2-EGFP positive and negative cell population were nearly 100%, they were also confirmed by the following microscopy analysis (S1A Fig).

### Bulk RNA-seq library preparation and sequencing

Ebf2-EGFP positive and negative cells were dissociated from the cerebral cortex and collected by FACS on E11.5, E13.5 and E15.5 stages, respectively. Approximately 12 Ebf2-EGFP positive embryos from three mice were used in each experiment at each stage and 10 million cells for each sample were collected with two biological replicates. Total RNA was extracted using QIAGEN RNeasy Mini Kits and processed by Sera-Mag Oligo (dT) beads (Thermo Scientific), libraries were prepared using the NEB Next mRNA Sample PreP Master Mix Set 1. All these experiments were performed according to the manufacturer’s instruction. Samples were sequenced on Illumina HiSeq 2000 using 100 base paired-end reads.

### Mapping and transcripts assembling

Average 60 million paired-end reads for each sample were obtained by HiSeq2000, then were mapped to the mouse genome build mm10 using Tophat v2.0.8b with about 85% mapping ratio [83]. To measure gene or lncRNA expression level, we calculated fragment per kilobase per million (FPKM) using Cufflinks v2.2.0 [84] based on the annotations for protein-coding genes (downloaded from UCSC, mm10, archive-2013-03-06-15-06-02) and known lncRNAs (lincRNAs and antisense lncRNAs, downloaded from ENSEMBL Release M6, 201507) [85]. Cuffdiff was used to identify differentially expressed genes between every two conditions (with 2 biological replicates) (FDR < 0.05 and fold change > 2 as cutoff) [86].

### scRNA-seq library preparation, sequencing and analysis

Ebf2-EGFP positive cells from E15.5 were dissociated and harvested by FACS, about eight embryos from two timed-pregnant mice were used in the experiments. Approximately 3000 cells were processed for downstream reverse transcription, cDNA synthesis and library preparation according to 10× Genomics Chromium Single Cell protocol. Samples were sequenced on Illumina HiSeq2500 platform using 150 base paired-end reads. Sequencing reads were analyzed by Cell Ranger (v2.0.0). The gene expression datasets were analyzed by Seurat (v2.0) and Monocle package.

### Primary neural cell culture assays and lentiviral overexpression

Embryonic cortical tissues were dissected and dissociated into single cells and cultured as previously described [29,87]. NSCs and lentivirus were added together in the adherent culture assay in serum-free N-2/B-27/DMEM medium with 10 ng/ml FGF2 (Life Tech) on poly-L-lysine (PLL, Sigma) pre-coated plates. Lentivirus infected cells were labeled by fluorescent protein GFP or RFP, and were used 3 days after plating for qPCR experiments (Figs 1F, 3F, S1F and S3B), 5 days for neural stem cells differentiation assays (Fig 4A) as described previously [87–89]. Ebf2 and Foxg1 lentiviral shRNAs were constructed as previously described [19,29]. Overexpression of lncRNA were constructed by cloning into a pLVX-pCAG-CDS-pUBC-GFP/RFP vector. These vectors were packaged into lentivirus in 293FT cells.

### Tissue collection and RNA *in situ* hybridization

Mouse brains at various developmental stages (E11.5, E13.5, E15.5) were collected and fixed in 4% paraformaldehyde (PFA) at 4°C for 4h. After briefly rinsing in PBS, brains were dehydrated in 30% sucrose in PBS at 4°C until the time they sank, and embedded in OCT (Sakura Finetek, Torrance, CA). Frozen brains were cryosectioned to 16 μm and mounted to coverslip. *In situ* hybridization was performed according to instructions as previously described [90,91]. Digoxigenin-labeled RNA probes were designed based on lncRNA sequences determined by Cufflinks and Ensembl. Images were taken on a Zeiss Slide Scanner (Axio Scan. Z1) fluorescent microscope.

### Chromatin-immunoprecipitation and sequencing (ChIP-Seq)

ChIP assays were performed using mouse Ebf2-EGFP positive cells dissociated at E11.5, E13.5, E15.5 and Ebf2-EGFP negative cells at E11.5, with antibodies against H3K27ac (Ab4729, Abcam) and H3K27me3 (Ab6002, Abcam) as previously described [92]. Briefly, Ebf2-EGFP positive and negative cells (~1–5 million cells) were sorted and collected from FACS (BD Influx), and fixed by 0.4% (v/v) formaldehyde at room temperature (RT) for 10 min. The reaction was stopped by adding 0.125 M glycine for 5 min. After rinsing cells three times with cold PBS, the nuclei were resuspended in Nuclei Lysis Buffer (50 mM Tri-Cl, pH8.1, 10 mM EDTA, 1% SDS, 1 mM DTT, 1 mM PMSF and Sigma protease inhibitor cocktail, RT) and incubated on ice for 10 min. Nuclear suspension were sonicated 6” on, 15” off, 20 cycles (power set at 25%) on ice. The fragmented chromatin was diluted for 5 folds in ChIP Dilution Buffer (16.7 mM Tri-Cl, pH8.1, 167 mM NaCl, 1.2 mM EDTA, 1.1% Triton X-100, 1 mM PMSF, and Sigma protease inhibitor cocktail). Then 5 μg IP antibody was added and incubated overnight at 4°C. Chromatin-protein complex was pulled down with Dynabeads protein G (Cat#10004D, Invitrogen) for 2h at 4°C with rotation and followed by 5 sequential washes as the following: 1. low salt wash buffer (20 mM Tri-Cl, pH8.1, 150 mM NaCl, 1 mM EDTA, 1% Triton X-100, 0.1% SDS, 0.1% Na-deoxycholate); 2. high salt wash buffer (20 mM Tri-Cl, pH8.1, 500 mM NaCl, 1 mM EDTA, 1% Triton X-100, 0.1% SDS, 0.1% Na-deoxycholate); 3. LiCl wash buffer (10 mM Tri-Cl, pH8.1, 250 mM LiCl, 1 mM EDTA, 0.5% NP-40, 0.5% Deoxycholic acid (Na salt)); and the last two washes with TE buffer (10 mM Tri-Cl, pH8.1, 1 mM EDTA). Immunoprecipitated complex was eluted from the antibody in elution buffer (1% SDS, 50 mM TrisCl (pH 8.1), 1 mM EDTA) by vortexing 10-15s once at 65°C for 20min and once at RT. Eluted chromatins were incubated at 65°C overnight for reverse crosslinks and chromatin fragmented purification with PCR column purification. ChIP-Seq libraries were prepared using NEB Next Ultra II ChIP-Seq Library Prep Kit (Cat#E7645S & Cat#7335S, NEB) according to the manufacturer’s instructions. ChIP-seq data were aligned to mouse genome mm10, performing Bowtie v. 1.1.2 on the Galaxy platform with the following options: -v 3 –M 1–5 15. Resulting output SAM files were converted to BAM format, and finally converted to BED files. Peaks were called using MACS (Model-Based Analysis of ChIP-Seq), with a p-value cutting off of 1e-5.

## Supporting information

S1 Fig Characterization of Ebf2-EGFP+ cell-enriched genes in the developing mouse forebrain. (A) Phase and fluorescent images of Ebf2-EGFP+ cells and Ebf2-EGFP- cells after FACS at Day 0 and Day 4 in vitro culture showing the purity and growth of sorted and unsorted cells (scale bar, 25 μm). (B) Sample to sample Pearson’s correlation (top-right quadrants) and pairwise comparison (bottom-left quadrants) of normalized gene expression between the two biological replicates and cell types. (C-D) qRT-PCR and endogenous expression patterns (*In situ* hybridization data from Allen Brain Atlas) to validate the RNA-seq results at different embryonic stages. CR neuron molecular markers as *Calb2*, *Ebf2*, *Tbr1*, and CR neuron related genes as *Ebf1*, *Ebf3*, *Foxg1*, and newly identified CR gene *Nhlh2* were selected for validation. (E-F) qRT-PCR analysis of CR-specific genes in primary neural cell culture assay after lentiviral transduction of control (H1) or *Ebf2*-knockdown (E), or *Foxg1*-knockdown (F). Data represent mean ± SEM (n = 3 independent experiments, **P<0.01, *P<0.05, T test).
(TIF)

S2 Fig Annotation of temporal dynamic genes. (A) Gene Ontology analysis of selected genes with distinct temporal dynamic expression pattern. (B) The qRT-PCR validation of DEGs from temporal dynamic expression pattern analysis (y axis = Fold Change of Ebf2-EGFP+ cells compare to Ebf2-EGFP- cells). Data represent mean (n = 3 independent experiments).
(TIF)

S3 Fig Functional prediction of CR-specific DElncRNAs. (A) qRT-PCR analysis to validate the RNA-seq results at three embryonic stages. CR-specific lncRNAs *Ln-CR5* and *Ln-CR6* were selected for validation. Data represent mean ± SEM (n = 3 independent experiments). (B) qRT-PCR analysis of CR-specific lncRNAs in primary neural cell culture assay after lentiviral transduction of control (H1) or *Foxg1*-knockdown. Data represent mean ± SEM (n = 4 independent experiments, **P<0.01, *P<0.05, T test). (C) *In situ* hybridization showing no expression in CR-specific lncRNA *Ln-CR2* negative control (sense probe) at E11.5, E13.5 and E15.5, respectively. Scale bar, 900 μm. (D) Immunostaining of E15.5 Ebf2-EGFP+ brain sections shown nearly all (98.67%) Ebf2+ cells were Reln+ cells. RELN (red), GFP (green), DAPI (blue) for Reln+ CR neurons, Ebf2+ CR neurons, and nucleus, respectively. Scale bar, 50 μm.
(TIF)

S4 Fig Functional validation of CR-specific lncRNAs *In Vitro* and *Ex Vivo*. (A) Immunostaining of neural cells treated with control (CAG-control) or overexpression lentiviruses for CR-specific lncRNAs *Ln-CR2* (CAG-*Ln-CR2*) and *Ln-CR3* (CAG-*Ln-CR3*) after in vitro culture for 5 days. RELN (red), TUJ1 (cyan), or DAPI (blue) for CR neurons, neurons, and nucleus, respectively. Scale bar, 50 μm. (B-E) Overexpression of CR-specific lncRNAs *Ln-CR2* and *Ln-CR3* increased CR neuron number, promoted NSC differentiation and generated more neuron-like small cell clones. All the experiments were performed 4 times and were counted 8 randomly selected microscopy fields each time. Data represented mean ± SEM. p values indicated were calculated by Student’s t test (unpaired), *p<0.05. (F) Quantification of process branch depth, branch levels, total dendrites length and soma size of CR neurons after overexpression of CR-specific lncRNAs *Ln-CR2* and *Ln-CR3* compared to control. All the experiments were performed 3 times and were counted 8 randomly selected microscopy fields each time. Data represented mean ± SEM. p values indicated were calculated by Student’s t test (unpaired), *p<0.05. (G) Comparison of dendritic spine, filopodia and processes of cultured cortical wholemounts overexpressing *Ln-CR1* (CAG-*Ln-CR1*), *Ln-CR2* (CAG-*Ln-CR2*) and *Ln-CR3* (CAG-*Ln-CR3*) to wholemounts expressing CAG-control. 10 days culture. Scale bar, 15 μm. (H) Quantification of CR neuron processes branch depth, branch levels and total dendrites length after overexpression of CR-specific lncRNAs, and compared to control group. All the experiments were performed 3 times and were counted 4 randomly selected microscopy fields each time. Data represent mean ± SEM. p values indicated were calculated by Student’s t test (unpaired), **p<0.01, *p<0.05. (I) Quantification of CR neuron molecular markers as *Reln*, *Ebf2*, and *Calb2* at 72h after lentiviral transduction in the NSC culture assay comparing the effect of overexpression of CR-specific lncRNAs *Ln-CR*2 and *Ln-CR*3 to control. Data represented mean ± SEM (**p<0.01, *p<0.05, n>3, Student’s T test).
(TIF)

S5 Fig Quality control and pseudotime lineage reconstruction analysis of single cell RNA-seq. (A) Violin plots shows genes, UMIs and mitochondrion ratio in scRNA-seq. Left, violin plot shows detected gene number in all sample cells. X-axis is sample name; Left, Y-axis is gene numbers detected in a single cell; Middle, Y-axis is UMIs detected in a single cell; Right, Y-axis is mitochondrion number detected in a single cell. (B) Scatter diagram of mitochondrion ratio, gene numbers compared to UMI numbers, respectively. X-axis is UMI numbers; Left, Y-axis is mitochondrion ratio detected in a single cell; Right, Y-axis is gene number detected in a single cell. (C) Scatter diagram of all detected genes dispersion. X-axis is average expression of a gene in all cells and Y-axis is dispersion value of that gene. (D) t-SNE clustering plot visualization of highly expressed molecular markers of each cell subpopulation. Each dot represents a single cell. Dot color represents expression level (from grey to purple, expression levels from low to high). (E) Visualization of typical CR origins as cortical hem, septum and ventral pallium derived gene *Lhx*5, VZ derived CR gene *Emx2* expression level across the eight major cell subpopulations using t-SNE clustering. (F) Pseudotime trajectories of E15.5 Ebf2-EGFP+ cells reconstructed by Monocle. Pseudotime points are indicated by colors and cell clusters are indicated by sequential numbers in the black circle. Dot color represents pseudotime points (from black to blue, pseudotime points from early to late). (G) Expression level dynamics of three developmental states genes as C157, C0346 and C2 coding genes and lncRNAs are shown across pseudotime trajectories. (H) The distribution of ZIC2 targeting genes across C157, C0346 and C2. (I) Violin plots showing *Foxg1* expression level across the eight cell subpopulations. Each dot represents a single cell.
(TIF)

S6 Fig Distinct H3K27ac and H3K27me3 chromatin signatures in Ebf2-EGFP+ cells. (A-B) Average profile of H3K27ac (A) or H3K27me3 (B) binding at called ChIP-seq peaks comparing the two biological replicates. (C-H) Quantitative data showing genomic features of H3K27ac and H3K27me3-bound genes in Ebf2-EGFP+ cells (C, D), E11.5 NPCs (E, F) and E11.5 Ebf2-EGFP+ cells (G, H). (I) Comparison of Ebf2-EGFP+ cell-enriched gene numbers with H3K27ac (red) and H3K27me3 (blue) bindings in Ebf2-EGFP+ cells. (J) Venn diagram showing overlapped genes with TSS H3K27ac bindings from E11.5 to E15.5, and C2 genes (Fig 1E) from single cell analysis. (K) The number of DEGs that stably lowly expressed (FPKM<1) from E11.5 to E15.5 with TSS or distal region H3K27ac, H3K27me3 occupancies in Ebf2-EGFP+ cells, respectively. The percentage indicate the proportion of all E11.5 to E15.5 low expressed DEGs (with and without histone modification). (L) The TSS regions of CR-specific genes *Sncb* showing bivalent in NPCs and becoming more activated with monovalent H3K27ac in Ebf2-EGFP+ cells from E11.5 to E15.5, its corresponding expression repressed in E11.5 Ebf2-EGFP- cells, and increased in Ebf2-EGFP+ cells from E11.5 to E13.5, while decreased in E15.5 as less H3K27ac enrichments. CR-specific lncRNAs *Ln-CR5* demonstrate a similar pattern of H3K27ac and H3K27me3 histone modification during early embryonic development.
(TIF)

S1 Table DEGs, DEGs enriched in Ebf2-EGFP+ cell population, CR-specific DEGs, CR-specific DEGs specifically expressed in layer 1. (XLSX)

S2 Table GO terms related to the Fig 2A–2F. (XLSX)

S3 Table 26 gene categories showing temporal dynamic gene expression pattern, related to Fig 2G. (XLSX)

S4 Table DElncRNAs, DElncRNAs enriched in Ebf2-EGFP+ cell population, CR-specific DElncRNAs. (XLSX)

S5 Table Key transcription factors interacting genes distributed across C157, C0346 and C2, separately. (XLSX)

S1 Text Supplemental Methods. (DOCX)
