# Supplementary figures and images for: Integrative genomic analysis of early neurogenesis reveals a temporal genetic program for differentiation and specification of preplate and Cajal-Retzius neurons
Source: PLoS Genet. 2021 Mar 24;17(3):e1009355. doi: 10.1371/journal.pgen.1009355 (PMC7990179; doi:10.1371/journal.pgen.1009355)

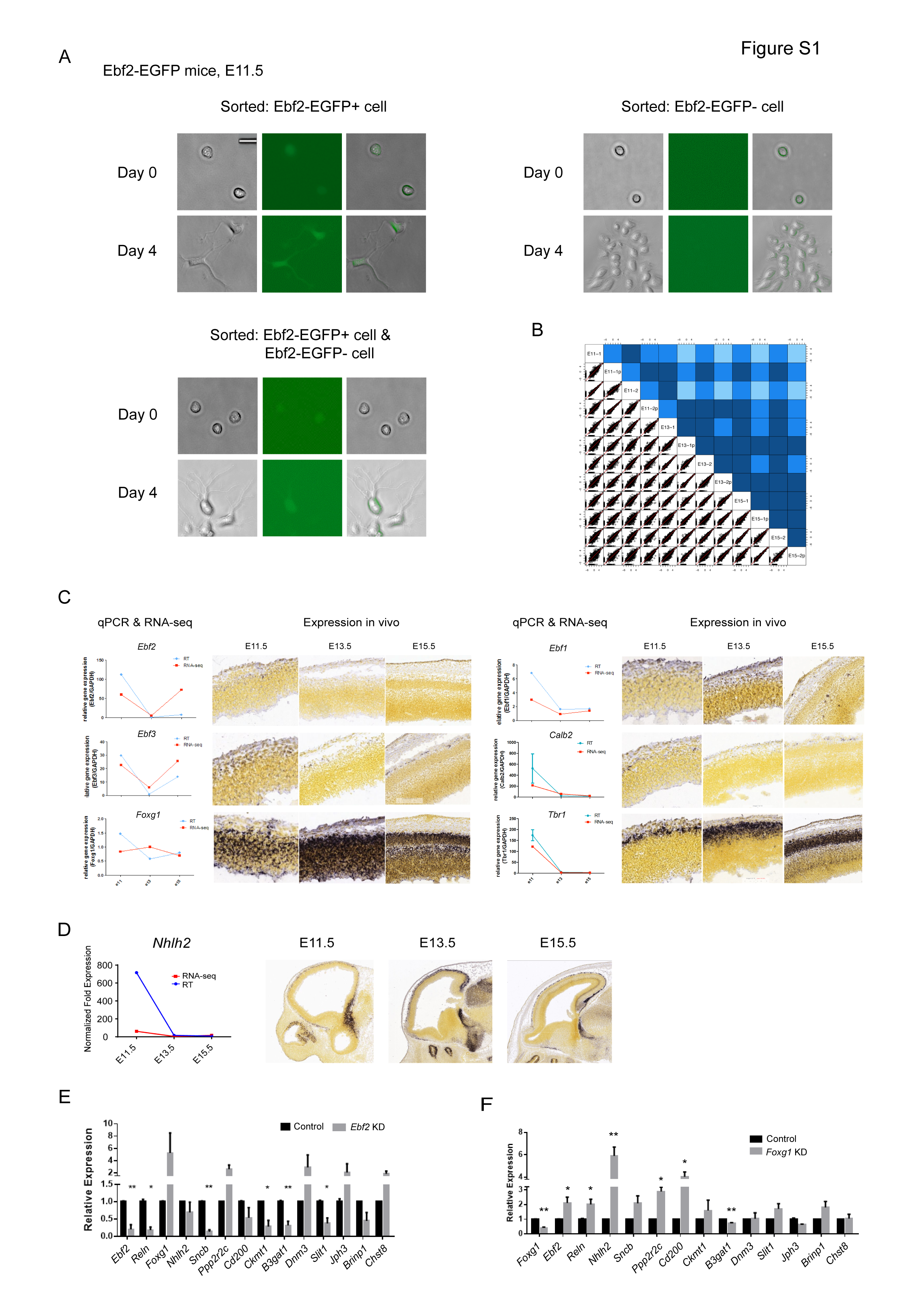

Supplement: S1 Fig — (A) Phase and fluorescent images of Ebf2-EGFP+ cells and Ebf2-EGFP- cells after FACS at Day 0 and Day 4 in vitro culture showing the purity and growth of sorted and unsorted cells (scale bar, 25 μm). (B) Sample to sample Pearson’s correlation (top-right quadrants) and pairwise comparison (bottom-left quadrants) of normalized gene expression between the two biological replicates and cell types. (C-D) qRT-PCR and endogenous expression patterns (In situ hybridization data from Allen Brain Atlas) to validate the RNA-seq results at different embryonic stages. CR neuron molecular markers as Calb2, Ebf2, Tbr1, and CR neuron related genes as Ebf1, Ebf3, Foxg1, and newly identified CR gene Nhlh2 were selected for validation. (E-F) qRT-PCR analysis of CR-specific genes in primary neural cell culture assay after lentiviral transduction of control (H1) or Ebf2-knockdown (E), or Foxg1-knockdown (F). Data represent mean ± SEM (n = 3 independent experiments, **P<0.01, *P<0.05, T test). (TIF) [file pgen.1009355.s001.tif]

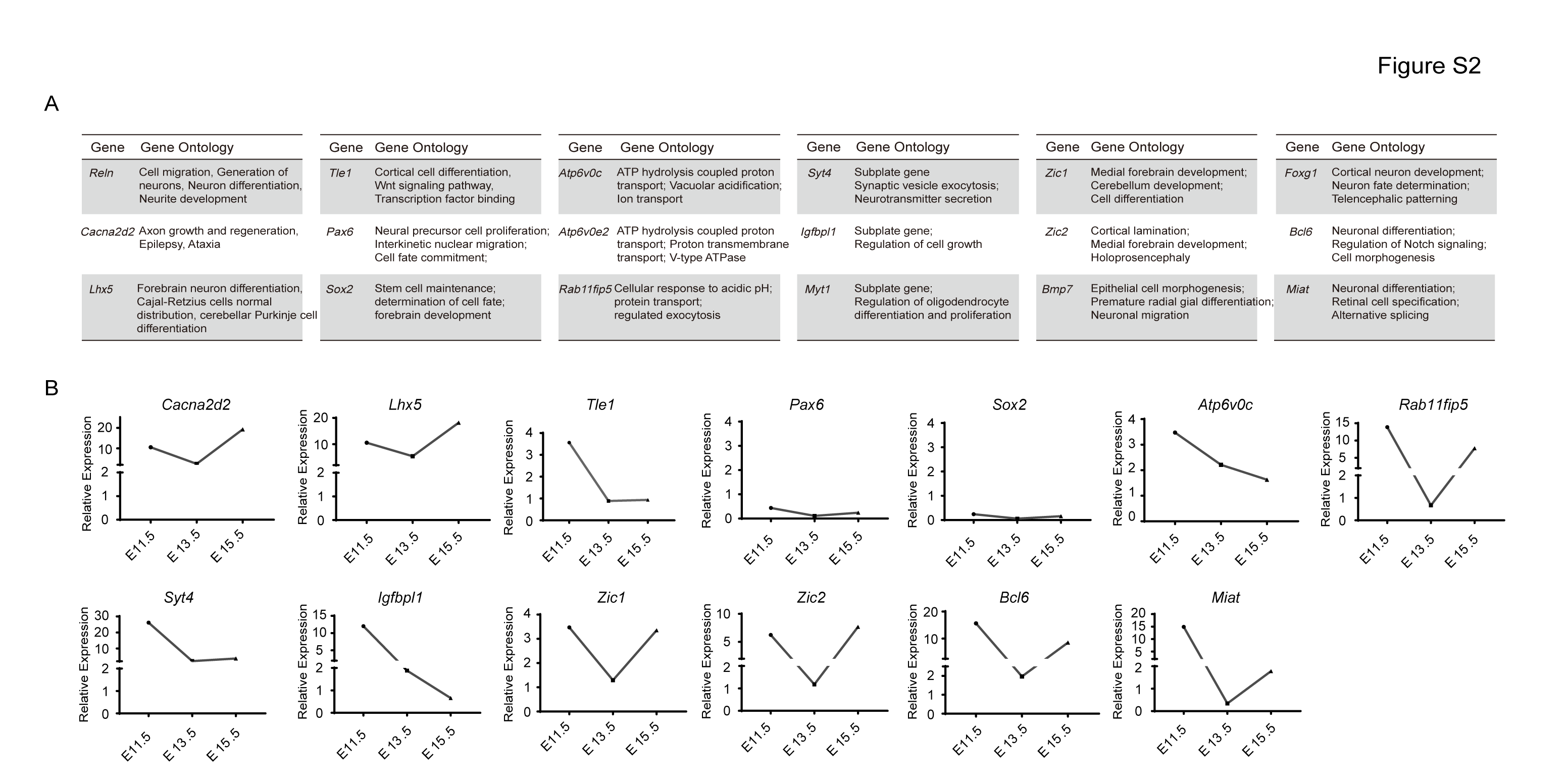

Supplement: S2 Fig — (A) Gene Ontology analysis of selected genes with distinct temporal dynamic expression pattern. (B) The qRT-PCR validation of DEGs from temporal dynamic expression pattern analysis (y axis = Fold Change of Ebf2-EGFP+ cells compare to Ebf2-EGFP- cells). Data represent mean (n = 3 independent experiments). (TIF) [file pgen.1009355.s002.tif]

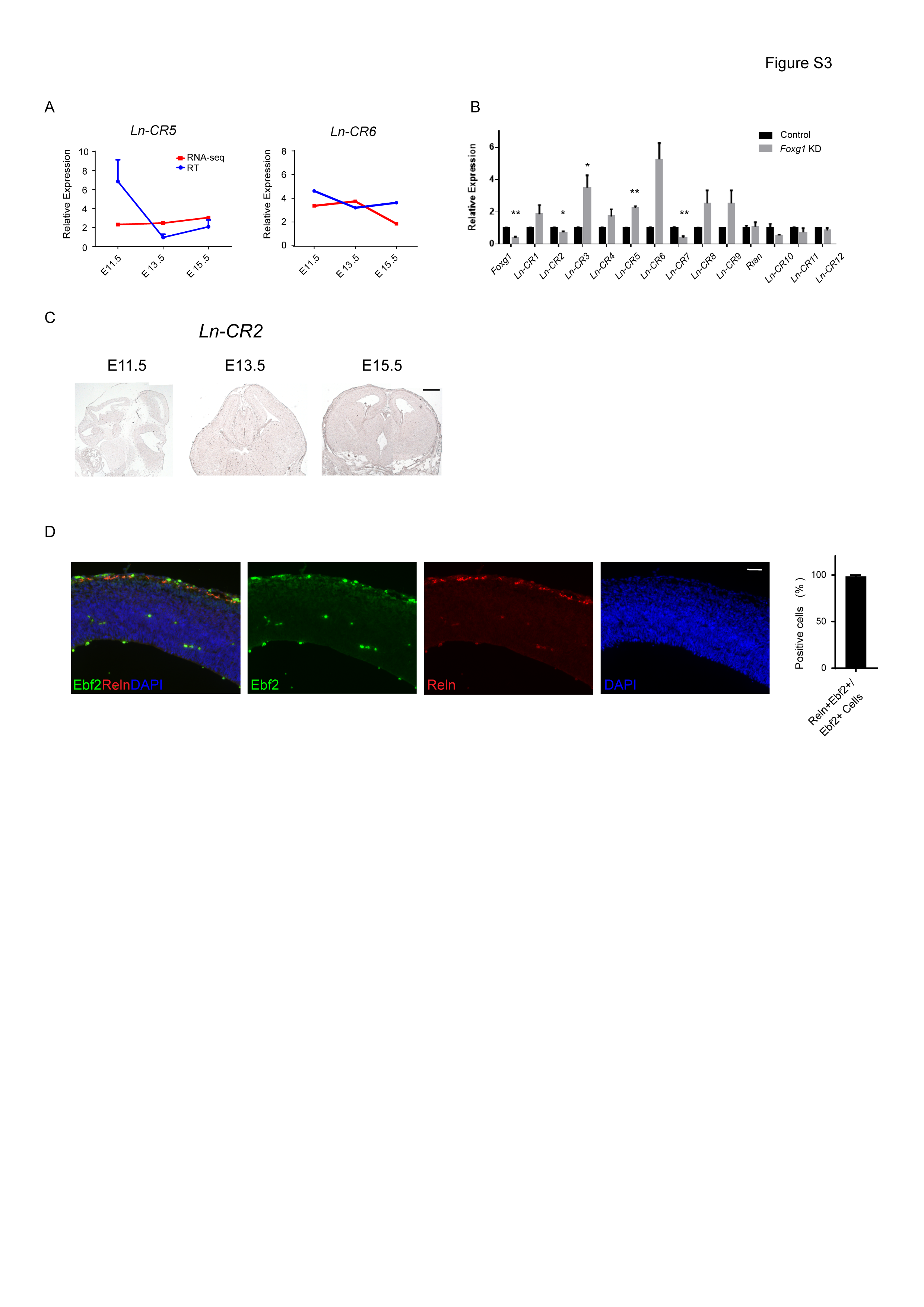

Supplement: S3 Fig — (A) qRT-PCR analysis to validate the RNA-seq results at three embryonic stages. CR-specific lncRNAs Ln-CR5 and Ln-CR6 were selected for validation. Data represent mean ± SEM (n = 3 independent experiments). (B) qRT-PCR analysis of CR-specific lncRNAs in primary neural cell culture assay after lentiviral transduction of control (H1) or Foxg1-knockdown. Data represent mean ± SEM (n = 4 independent experiments, **P<0.01, *P<0.05, T test). (C) In situ hybridization showing no expression in CR-specific lncRNA Ln-CR2 negative control (sense probe) at E11.5, E13.5 and E15.5, respectively. Scale bar, 900 μm. (D) Immunostaining of E15.5 Ebf2-EGFP+ brain sections shown nearly all (98.67%) Ebf2+ cells were Reln+ cells. RELN (red), GFP (green), DAPI (blue) for Reln+ CR neurons, Ebf2+ CR neurons, and nucleus, respectively. Scale bar, 50 μm. (TIF) [file pgen.1009355.s003.tif]

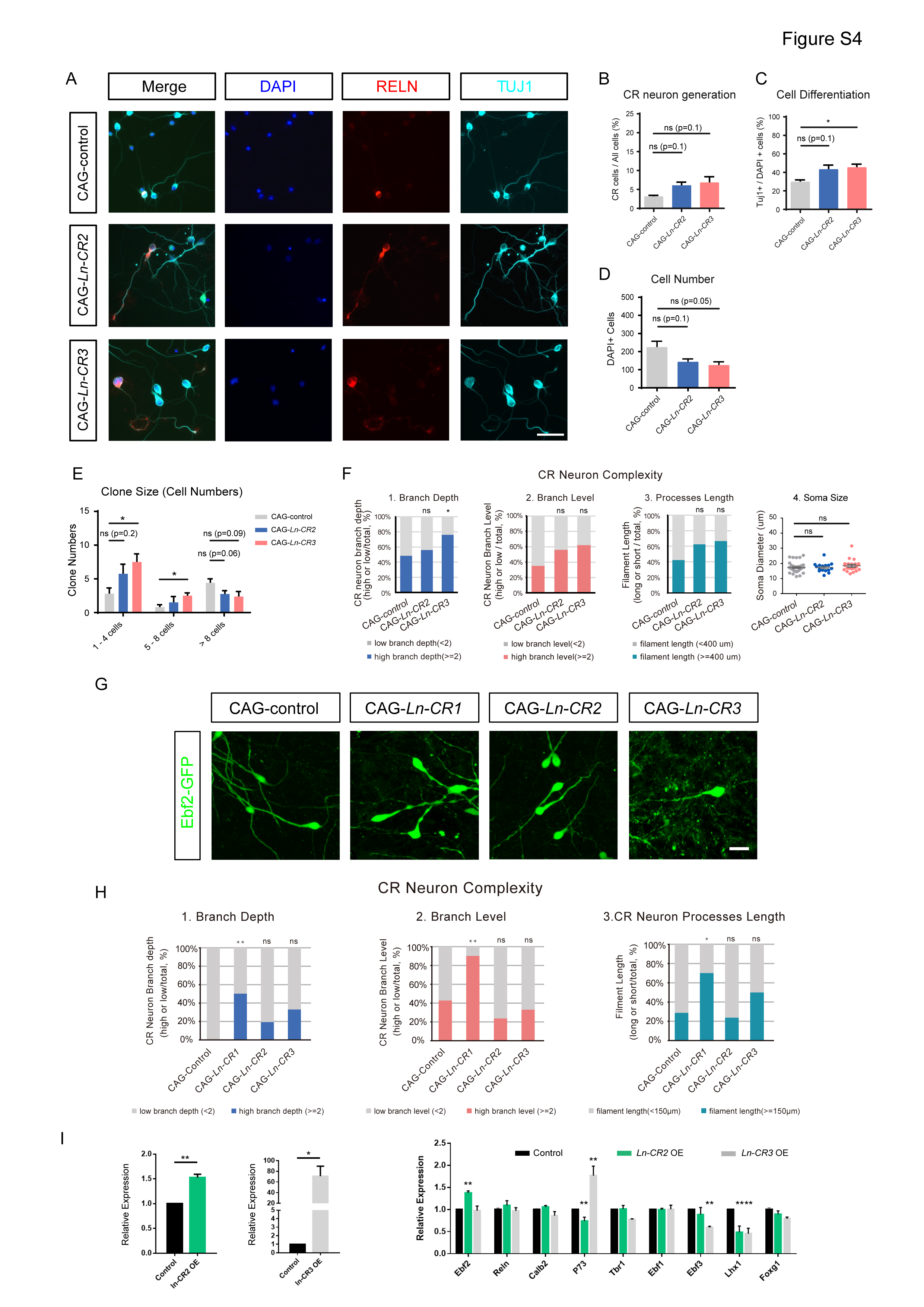

Supplement: S4 Fig — (A) Immunostaining of neural cells treated with control (CAG-control) or overexpression lentiviruses for CR-specific lncRNAs Ln-CR2 (CAG-Ln-CR2) and Ln-CR3 (CAG-Ln-CR3) after in vitro culture for 5 days. RELN (red), TUJ1 (cyan), or DAPI (blue) for CR neurons, neurons, and nucleus, respectively. Scale bar, 50 μm. (B-E) Overexpression of CR-specific lncRNAs Ln-CR2 and Ln-CR3 increased CR neuron number, promoted NSC differentiation and generated more neuron-like small cell clones. All the experiments were performed 4 times and were counted 8 randomly selected microscopy fields each time. Data represented mean ± SEM. p values indicated were calculated by Student’s t test (unpaired), *p<0.05. (F) Quantification of process branch depth, branch levels, total dendrites length and soma size of CR neurons after overexpression of CR-specific lncRNAs Ln-CR2 and Ln-CR3 compared to control. All the experiments were performed 3 times and were counted 8 randomly selected microscopy fields each time. Data represented mean ± SEM. p values indicated were calculated by Student’s t test (unpaired), *p<0.05. (G) Comparison of dendritic spine, filopodia and processes of cultured cortical wholemounts overexpressing Ln-CR1 (CAG-Ln-CR1), Ln-CR2 (CAG-Ln-CR2) and Ln-CR3 (CAG-Ln-CR3) to wholemounts expressing CAG-control. 10 days culture. Scale bar, 15 μm. (H) Quantification of CR neuron processes branch depth, branch levels and total dendrites length after overexpression of CR-specific lncRNAs, and compared to control group. All the experiments were performed 3 times and were counted 4 randomly selected microscopy fields each time. Data represent mean ± SEM. p values indicated were calculated by Student’s t test (unpaired), **p<0.01, *p<0.05. (I) Quantification of CR neuron molecular markers as Reln, Ebf2, and Calb2 at 72h after lentiviral transduction in the NSC culture assay comparing the effect of overexpression of CR-specific lncRNAs Ln-CR2 and Ln-CR3 to control. Data represented mean ± SE [file pgen.1009355.s004.tif]

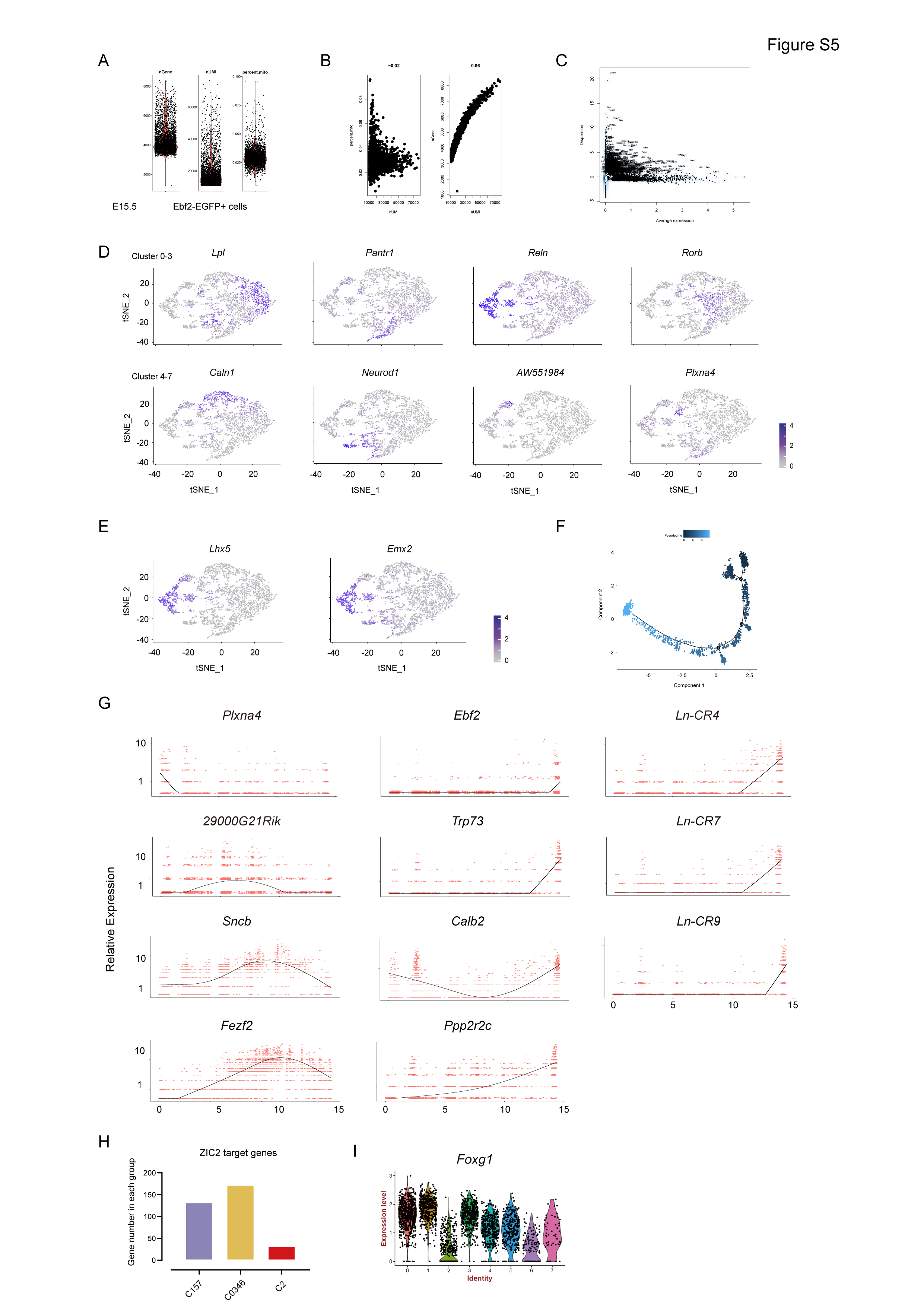

Supplement: S5 Fig — (A) Violin plots shows genes, UMIs and mitochondrion ratio in scRNA-seq. Left, violin plot shows detected gene number in all sample cells. X-axis is sample name; Left, Y-axis is gene numbers detected in a single cell; Middle, Y-axis is UMIs detected in a single cell; Right, Y-axis is mitochondrion number detected in a single cell. (B) Scatter diagram of mitochondrion ratio, gene numbers compared to UMI numbers, respectively. X-axis is UMI numbers; Left, Y-axis is mitochondrion ratio detected in a single cell; Right, Y-axis is gene number detected in a single cell. (C) Scatter diagram of all detected genes dispersion. X-axis is average expression of a gene in all cells and Y-axis is dispersion value of that gene. (D) t-SNE clustering plot visualization of highly expressed molecular markers of each cell subpopulation. Each dot represents a single cell. Dot color represents expression level (from grey to purple, expression levels from low to high). (E) Visualization of typical CR origins as cortical hem, septum and ventral pallium derived gene Lhx5, VZ derived CR gene Emx2 expression level across the eight major cell subpopulations using t-SNE clustering. (F) Pseudotime trajectories of E15.5 Ebf2-EGFP+ cells reconstructed by Monocle. Pseudotime points are indicated by colors and cell clusters are indicated by sequential numbers in the black circle. Dot color represents pseudotime points (from black to blue, pseudotime points from early to late). (G) Expression level dynamics of three developmental states genes as C157, C0346 and C2 coding genes and lncRNAs are shown across pseudotime trajectories. (H) The distribution of ZIC2 targeting genes across C157, C0346 and C2. (I) Violin plots showing Foxg1 expression level across the eight cell subpopulations. Each dot represents a single cell. (TIF) [file pgen.1009355.s005.tif]

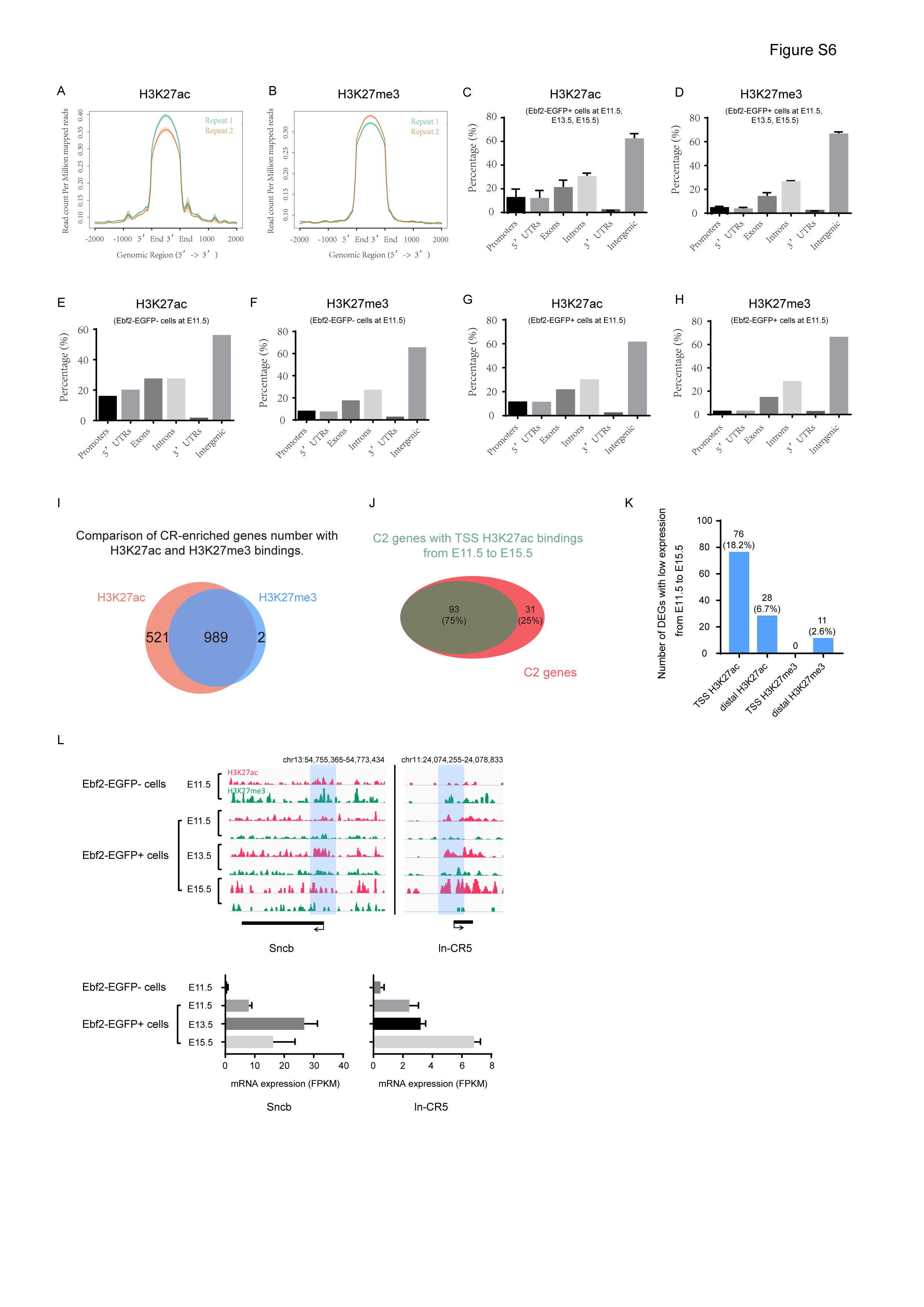

Supplement: S6 Fig — (A-B) Average profile of H3K27ac (A) or H3K27me3 (B) binding at called ChIP-seq peaks comparing the two biological replicates. (C-H) Quantitative data showing genomic features of H3K27ac and H3K27me3-bound genes in Ebf2-EGFP+ cells (C, D), E11.5 NPCs (E, F) and E11.5 Ebf2-EGFP+ cells (G, H). (I) Comparison of Ebf2-EGFP+ cell-enriched gene numbers with H3K27ac (red) and H3K27me3 (blue) bindings in Ebf2-EGFP+ cells. (J) Venn diagram showing overlapped genes with TSS H3K27ac bindings from E11.5 to E15.5, and C2 genes (Fig 1E) from single cell analysis. (K) The number of DEGs that stably lowly expressed (FPKM<1) from E11.5 to E15.5 with TSS or distal region H3K27ac, H3K27me3 occupancies in Ebf2-EGFP+ cells, respectively. The percentage indicate the proportion of all E11.5 to E15.5 low expressed DEGs (with and without histone modification). (L) The TSS regions of CR-specific genes Sncb showing bivalent in NPCs and becoming more activated with monovalent H3K27ac in Ebf2-EGFP+ cells from E11.5 to E15.5, its corresponding expression repressed in E11.5 Ebf2-EGFP- cells, and increased in Ebf2-EGFP+ cells from E11.5 to E13.5, while decreased in E15.5 as less H3K27ac enrichments. CR-specific lncRNAs Ln-CR5 demonstrate a similar pattern of H3K27ac and H3K27me3 histone modification during early embryonic development. (TIF) [file pgen.1009355.s006.tif]
