# Supplementary material for: Integrative genomic analysis of early neurogenesis reveals a temporal genetic program for differentiation and specification of preplate and Cajal-Retzius neurons
Source: PLoS Genet. 2021 Mar 24;17(3):e1009355. doi: 10.1371/journal.pgen.1009355 (PMC7990179; doi:10.1371/journal.pgen.1009355)
Supplement: S1 Text — (DOCX) [file pgen.1009355.s012.docx]

**S1 Text. Supplemental Methods**

**Clustering analysis and functional enrichment analysis**

K-means clustering algorithm based on Euclidean distance was used to distinguish different gene expression patterns, which was visualized by Java TreeView software [1]. Then, we used findGO.pl program in Homer to perform functional enrichment (GO annotation, KEGG and Wiki Pathway) analysis for each gene set with different expression patterns [2].

**Functional analysis of lncRNAs**

To identify the function of lncRNAs, we calculated the expression correlation of lncRNAs with coding genes. We assumed that co-expressed genes were more likely to be correlated in function. Based on expression profiles in lncRNAs and protein coding genes, we calculated the correlation between them. FPKM of genes and lncRNAs from RNA-seq data sets were used to calculate the correlation matrix. Before calculating the correlation matrix, we filtered out some low-expressed genes and lncRNAs for the accuracy of further characterization. For protein coding genes with FPKM ranked in top 75% in at least one data set were retained. For lncRNAs, the filtering processes were as follows: (1) expressed (FPKM>0) in at least 1 condition, (2) FPKM ranked in top 75% in at least one dataset, (3) co-efficient of variation (cv) of FPKM ranked in top 75% in all ncRNA candidates. Cv represented for coefficient of variance, the standard deviation divided by mean. Cv showed the extent of variability in relation to mean of the population.

Correlations between gene-gene and gene-lncRNA were calculated. We chose to calculate Pearson correlation in the following analysis. The Pearson correlation coefficient was a measure of the linear correlation between two variables X and Y, giving a value between +1 and −1 inclusive, where 1 was total positive correlation, 0 was no correlation, and −1 was negative correlation. It was widely used in the sciences as a measure of the degree of linear dependence between two variables. Pearson's correlation coefficient between two variables was defined as the covariance of the two variables divided by the product of their standard deviations.


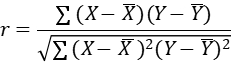
 (1)

The calculation of correlation was done by SciPy, a fundamental python library for scientific computing. The two-sided p-value for a hypothetical test whose null hypothesis was that two sets of data were uncorrelated. The p-value roughly indicated the probability of an uncorrelated system producing datasets that had a Pearson correlation at least as extreme as the one computed from these datasets.

Based on the assumption that functionally correlated genes were likely to co-express in various conditions, the functional prediction or annotation of lncRNAs could be performed. One of the most direct way was to look at the functional annotations of genes in its immediate neighborhood. For each lncRNAs, we chose top 1000 correlated genes to perform Gene Ontology analysis. We used a R package GOstats to perform GO analysis. The enriched GO term could be used to illustrate the possible function of lncRNAs.

**Quantitative RT-PCR**

Total RNA was isolated from purified Ebf2-EGFP positive or negative cells, extracted using Trizol reagent (Cat#15596018, Life Technologies), and reverse transcribed. qPCR was performed using PrimeScript RT regent Kit (Perfect Real Time, Cat#DRR047A, Takara). Gene expression levels were detected using a BioRad CFX384 Touch™ real-time PCR detection system (Bio-Rad Laboratories) and determined with ΔΔCt method, normalized to an internal control *Gapdh* mRNA for quantification. All reactions were replicated 3 times.

**In utero electroporation of cortical layer 1**

The E15.5 embryonic cortical layer 1 were electroporated as described previously [3]. Briefly, embryonic day 15.5 (E15.5) mice were anesthetized and placed on a heating pad. Then the uterus were carefully pulled out and injected with proper volume of plasmid mix (0.5-1µl/embryo, 2-3 µg/µl) into the gap under the skull and above the pial surface with a glass capillary. The Electroporator (CUY21VIVO-SQ; BEX) was set to five 50ms, 36V pulses with an interval of 999ms, and embryos’ heads were electroporated with a 5-mm electrode (LF650P5; BEX). After that, the embryos were placed back into their original location of abdominal cavity, and the abdomen wall and skin were closed. Then waited for a while until the mice recovered from surgery. For phenotype observation, the mice progenies were sacrificed on postnatal day 3.5 or 5.5 (P3.5 or P5.5), and both Ebf2-EGFP and RFP signals positive pups were selected for the following PFA fixation dealing and immunohistochemistry. Confocal images were taken using LSM780 (Zeiss) Laser Scanning System on a Zeiss microscope. At least three independent experiments, control group n=5, *ln-CR1* overexpression group n=3, 1-2 animals each experiment were analyzed for each condition. Finally control group n=8 mice, *ln-CR1* overexpression group n=5 mice.

**Wholemount culture neuronal processes analysis**

For neuronal processes and spine analysis, Ebf2-EGFP+ wholemounts of E15.5 cerebral cortex were infected by lenti-virus CAG-RFP, H1-RFP, or CAG-*Ln-CR*1, CAG-*Ln-CR*2, CAG-*Ln-CR*3. Next, wholemounts were cultured for 12 days in a 35℃ bioincubator. The culture medium was replaced every 3 days. After culture, wholemounts were fixed and stained with chicken anti-GFP (GFP-1010, Aves lab lnc.) and rat anti-chicken-Alexa488 (Jackson Immuno Research lab). Confocal microscopy was performed with a LSM780 (Zeiss) Laser Scanning System on a Zeiss microscope, and wholemount tissues were analyzed by taking 1µm serial optical sections with ZEN_2011_Lite_x64 software (Zeiss). Images were acquired using a 1024x1024 scan format with a 63x objective.

**Immunocytochemistry**

Cell culture media were removed, and cells were fixed in 4% (w/v) PFA for 30min at RT, rinsed by 1×PBS three times. Fixed cells were blocked in 5% (v/v) BSA with 0.3% (v/v) Triton X-100 in 1×PBS for 1h and rinsed by 1×PBS three times. Immunostainings were performed for 4h at RT or overnight at 4°C with rotation by incubating with primary antibodies, then washed by 1×PBS three times. Cells were incubated with secondary antibodies and DAPI diluted in 1×PBS for 1h in the dark, followed by washing with 1×PBS three times. Images were taken on Zeiss fluorescent microscopy (AxioVision). Primary antibodies used: GFP (GFP-1010, Aves lab lnc.), RELN (MAB5364, Millipore), TUJ1 (T8660, Sigma).

## SUPPLEMENTAL REFERENCES

1. Saldanha A. Java Treeview--extensible visualization of microarray data. Bioinformatics. 2004;20:3246-8.

2. Heinz S, Benner C, Spann N, Bertolino E, Lin YC, Laslo P, Cheng JX, Murre C, Singh H, C Glass. Simple combinations of lineage-determining transcription factors prime cis-regulatory elements required for macrophage and B cell identities. Mol Cell. 2010;38:576-89.

3. Levy R, Molina J, Danielpour M, B JJ. Neonatal pial surface electroporation. J Vis Exp. 2014:1-7.
